# An optimized deep-forest algorithm using a modified differential evolution optimization algorithm: A case of host-pathogen protein-protein interaction prediction

**DOI:** 10.1016/j.csbj.2025.01.020

**Published:** 2025-01-26

**Authors:** Jerry Emmanuel, Itunuoluwa Isewon, Jelili Oyelade

**Affiliations:** a Department of Computer and Information Sciences, Covenant University, Ota, Nigeria; b Covenant Applied Informatics and Communication African Centre of Excellence (CApIC-ACE), Nigeria; c Covenant University Bioinformatics Research (CUBRe), Nigeria

**Keywords:** Deep forest, Differential evolution, Optimization, Hyperparameter, Protein-protein interaction, *Plasmodium falciparum*

## Abstract

Deep Forest employs forest structures and leverages deep architecture to learn feature vector information adaptively. However, deep forest-based models have limitations such as manual hyperparameter optimization and time and memory usage inefficiencies. Bayesian optimization is a widely used model-based hyperparameter optimization method. Evolutionary algorithms such as Differential Evolution (DE) have recently been introduced to improve Bayesian optimization’s acquisition function. Despite its effectiveness, DE has a significant drawback as it relies on randomly selecting indices from the population of target vectors to construct donor vectors in search of optimal solutions. This randomness is ineffective, as suboptimal or redundant indices may be selected. Therefore, in this research we developed a modified differential evolution (DE) acquisition function for improved host-pathogen protein-protein interaction prediction. The modified DE introduces a weighted and adaptive donor vector technique that selects the best-fitted donor vectors as opposed to the random approach. This modified optimization approach was implemented in a deep forest model for automatic hyperparameter optimization. The performance of the optimized deep forest model was evaluated on human-*Plasmodium falciparum* protein sequence datasets using 10-fold cross-validation. The results were compared with standard optimization methods such as traditional Bayesian optimization, genetic algorithms, evolutionary strategies, and other machine learning models. The optimized model achieved an accuracy of 89.3 %, outperforming other models across all metrics, including a sensitivity of 85.4 % and a precision of 91.6 %. Additionally, the optimized model predicted seven novel host-pathogen interactions. Finally, the model was implemented as a web application which is accessible at http://dfh3pi.covenantuniversity.edu.ng.

## Highlights

• A modified differential evolution (DE) method was designed to improve the selection of hyperparameter configurations.
• The modified DE outperformed existing optimization algorithms, including genetic algorithms and evolutionary strategies.
• The modified DE demonstrated competitive time and memory efficiency in multiple experiments.
• The modified DE was used to optimize the deep forest model for predicting human-*Plasmodium falciparum* PPIs.
• The optimized DF was validated on customer churn and MNIST datasets and was later developed into a tool for PPI prediction.

## Graphical Abstract

**Figure:**
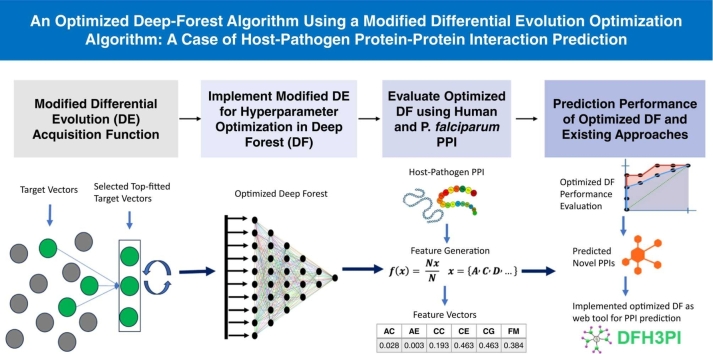

## 1 Introduction

Machine learning models offer extensive reconfigurability through their hyperparameters [1]. Unlike model parameters, which are internal and learned during training, hyperparameters are set based on domain knowledge or heuristics [2], [3]. They control various aspects of the learning process and significantly impact the model's complexity, behavior, and speed. Therefore, careful selection of hyperparameters is crucial to achieving optimal performance [4], [5]. Manually selecting the values of hyperparameters through trial and error is time-consuming, potentially biased, prone to errors, and often lacks computational reproducibility [6]. Automating the selection process for hyperparameters leads to developing more effective models. This process involves exploring different combinations of hyperparameters within the provided search space to determine which ones yield the best results. This process is known as hyperparameter optimization [7], [8].

Hyperparameter optimization is essential in machine learning for multiple reasons. Firstly, the process significantly impacts model performance to enhance accuracy and effectiveness. Additionally, it aids in preventing overfitting or underfitting by achieving an optimal balance between model complexity and data fitting. Hyperparameter optimization also ensures that models perform effectively on new, unseen data, achieving robustness and reliability. Optimization processes streamline resource utilization by efficiently finding optimal hyperparameters, thereby saving computational resources and minimizing the need for extensive trial and error [9], [10], [11], [12]. Moreover, as the volume and diversity of data continue to expand, optimizing hyperparameters becomes essential for ensuring that models can effectively process and analyze vast amounts of information, leading to more accurate predictions [13], [14], [15].

Some of the commonly used hyperparameter optimization methods include manual optimization, random search, and grid search [13], [15], [16]. However, due to a lack of knowledge-based decisions in these methods [3], [4], [13], [15], [16], model-based optimization methods such as Bayesian optimization, hyperband, Bayesian optimization and hyperband (BOHB), and population-based training (PBT) were developed [6], [17], [18]. Model-based hyperparameter optimization involves defining a search space, objective function, probabilistic model, acquisition function, and a history of evaluations. The search space represents the range of possible hyperparameters to explore [19], [20]. The objective function evaluates the performance of a set of hyperparameters by quantifying how well the model fits the data. A probabilistic model is used to predict the performance of different hyperparameters across the search space based on previous evaluations [2], [20]. The acquisition function leverages the probabilistic model to determine the next set of hyperparameters to evaluate, balancing the exploration of new regions and exploitation of known promising areas [11], [21]. The algorithm uses these components to iteratively update the surrogate model and select hyperparameters that improve the objective function's score. Most recently, evolutionary algorithms such as genetic algorithms, evolutionary strategies, and differential evolution have been employed to enhance further the process of generating candidate hyperparameter configurations in the acquisition functions of Bayesian optimization [11].

As highlighted, hyperparameter optimization impacts model accuracy and efficiency. One such model is deep forest, a robust framework that utilizes a cascade of decision tree ensembles [22], [23]. This ensemble-based approach has proven to enhance prediction accuracy and robustness, surpassing the performance of individual decision trees [24], [25]. Additionally, Deep Forest has demonstrated competitive results in addressing the challenges faced by traditional and deep neural network models, such as the need for large-scale datasets [26], [27], [28], the high computational demands of neural networks due to their architectural complexity [28], [29], and the challenges and inefficiencies of selecting optimal hyperparameters for different datasets [30], leading to multiple trials and increased computational cost [31], [32], [33], [34]. Furthermore, since its inception, deep forest-based models have been employed across a range of classification tasks, among which include SQL injection detection [35], industrial fault diagnosis [36], [37], cancer subtype classification [38], automobile predictive Maintenance [39], hyperspectral image (HSI) classification [40], [41], anti-cancer drug response classification [42], adaptive feature selection for COVID-19 classification using Chest computer tomography (CT) images [43], and building occupancy prediction [44]. Most recently, the deep forest model has also been employed for Host-Pathogen Protein-Protein Interaction (HPPPI) prediction [34], [45], [46] due to its incorporation of diverse classifiers to enhance performance and its adaptability to large and small datasets.

Despite the great potential and promising results obtained from its use, there are open problems in developing efficient deep forest models, as evident from literature. Several authors have reported that the hyperparameter values were obtained manually, and a limited range of hyperparameters were tested, which may not fully optimize the predictor’s performance. For instance, [47] reported that to derive the number of estimators for the improved intra-species Protein-Protein Interaction (PPI) predictor called Grained Cascade Forest (GcForest-PPI), the authors manually experimented with 2, 4, and 6 numbers of estimators in different experiments before concluding that 4 was the optimal number. Similarly, [34] reported to have used a computationally expensive grid search approach to determine the optimal hyperparameter values for a viral-host PPI predictor called Local Global Context-Aware Virus-Host Protein-Protein Interaction (LGCA-VHPPI). This highlights a common weakness in the computational expense associated with hyperparameter optimization methods, as validated by [48] in a study where a novel deep forest-based model for predicting activatory and inhibitory Drug Target Interactions (DTI) was developed.

Another recurring challenge is the need for improvement in the deep forest method itself, as indicated by [49], who reported that the Support Vector Machine (SVM) outperformed deep forest in terms of precision across experiments. Additionally, [48] noted that while the deep forest-based DTI predictor produced superior results, the prediction performance still required further improvement. [50] also reported suboptimal performance with the Long non-coding RNAs and Micro RNAs Interactions Deep Forest (LMI-DForest) method. Traditional deep forest and Extreme Gradient Boost (XGBoost) models recorded a better recall than LMI-DForest, indicating potential limitations in the approach to classifying long non-coding RNAs and miRNAs. Overall, the challenges and weaknesses of deep forest observed across these studies highlight common patterns, including the need for automating hyperparameter optimization, improving hyperparameter optimization methods, and further refinement of the deep forest method to improve its predictive capability over existing machine learning methods.

Furthermore, differential evolution as an acquisition function in Bayesian optimization is known for effectively exploring large search spaces. However, a significant drawback lies in its reliance on randomly selecting indices from the population of target vectors (initial or updated set of hyperparameter configurations) [11], [21]. These randomly chosen indices are then used to construct donor vectors, which guide the search process for better solutions. This randomness can lead to inefficiencies by selecting suboptimal or redundant indices.

Therefore, this study presents an optimized deep forest model that employs a modified differential evolution method to address the aforementioned challenges.

## 2 Materials and methods

### 2.1 Defining gold dataset

The first category of human-*Plasmodium falciparum* (human-PF) interaction used in this work has 1136 negative interactions and 1112 positive interactions. According to [51], the positive training set comprises structurally inferred and experimentally obtained interactions. According to the authors, a basic heuristic approach was employed due to the unavailability of gold standard negative training samples of non-interacting protein pairs. A negative training set, approximately the same size as the positive set, was created by randomly selecting pairs of parasite proteins and human proteins not included in the positive set [52], [53]. Secondly, another set of experimentally derived and structurally inferred protein interacting pairs used by Agamah [54] was also extracted from the research. This dataset comprises 456 interacting human-*Plasmodium falciparum* protein pairs, including their names and annotations. Thirdly, protein profiles of 5000 predicted non-interacting proteins of the human-*Plasmodium falciparum* network approach were extracted from the research conducted by [55].

### 2.2 Data preparation and preprocessing

**Fig. 1:**
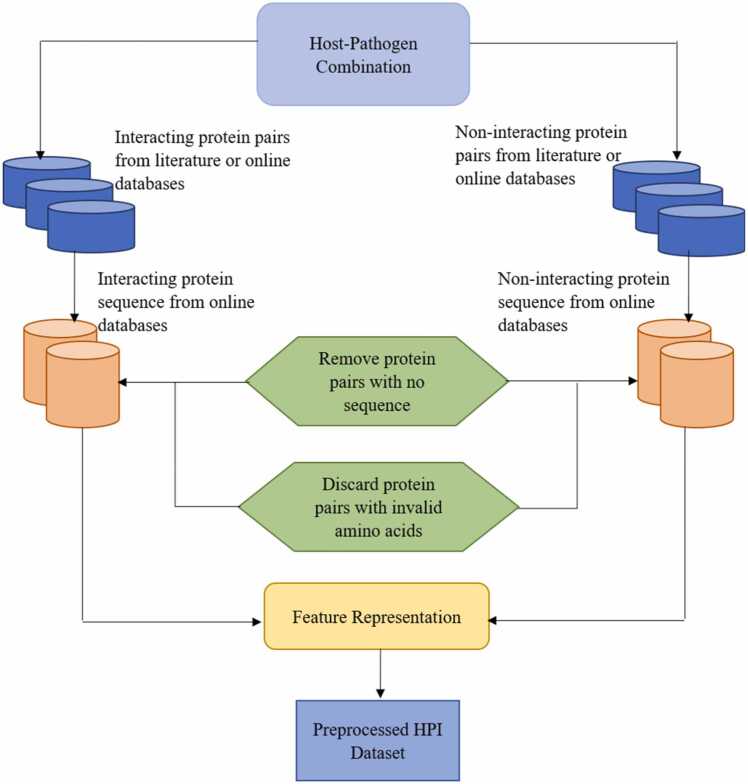
An illustration of the data preparation and preprocessing steps**.**

In the data preparation and preprocessing stage as illustrated in Fig. 1, the interacting protein pairs from human-PF combinations identified and retrieved in Section 2.1, were preprocessed to fit into the proposed model. After obtaining the protein names, the sequences for the identified human-*Plasmodium falciparum* proteins were sourced from online protein databases, such as UniProt [56], NCBI [57], and PlasmoDB [58]. Similarly, the amino acid sequences of the non-interacting proteins were also retrieved from online databases such as Negatome [59] and UniProt [56]. The protein sequences are a series of letters representing the amino acid composition in each protein. At this point, there were protein names whose amino acid sequences were not found in any of the online databases. As a result, any pairs with missing sequences for either the pathogen or host proteins were removed from the non-interaction and the interaction lists.

Similarly, a function was developed to scan through the amino acid sequence for each protein pair; any pair whose sequence contained an invalid amino acid letter was filtered out from the entire dataset. This ensures that only human-*Plasmodium falciparum* with valid amino acids were used in training and validating the developed model. Additionally, Table 1 shows the categories of datasets, including the number of interacting and non-interacting samples employed after the preprocessing stage. From the total of 1568 positive and 1136 negative PPI datasets, 806 interacting and 830 non-interacting pairs were extracted from the Wuchty [51] dataset, while the Vignali *et al.* [54] dataset contributed 24 interacting pairs. The total number of pairs used for training comprised 830 interacting and 830 non-interacting pairs, with 738 interacting and 306 non-interacting pairs remaining unused due to the absence or invalidity of protein profiles. For the validation set, the Agamah *et al.* [55] dataset provided a total of 4982 used pairs, with 18 interacting pairs remaining unused.

**Table 1:** Categories and number of samples used after preprocessing.

S/N	Human-*Plasmodium falciparum*		Interacting	Non-Interacting
		**Training Set**		
1	[51]		806	830
2	[54]		24	
	**Total used:**		**830**	**830**
	**Total unused:**		**738**	**306**
		**Validation Set**		
1	[55]**Used:**		4982	
	**Unused:**		18	

Furthermore, the protein sequences were grouped based on the similarity of the host (human) protein sequences, and each group was given a group ID. This was conducted to prevent overlapping of host-pathogen pairs during cross-validation, thereby preventing accuracy or performance evaluation inflation [46]. Across all training datasets, comprising 1660 non-interacting and interacting pairs, 834 group IDs were derived. Table 2 presents the group IDs and the corresponding number of protein sequences that share similar host sequences. For instance, Groups 77 and 122 each contain 10 interactions with identical host sequences. Detailed information on the protein sequence grouping and the algorithm is provided in the supplementary file S1 Algorithm 1.

**Table 2:** Group IDs and the corresponding number of protein sequences.

**Group IDs**	**Count**
583, 559, 573, 1, 591, 768, 760, 761, 764, 765, 766, 767, 770, 740, 772, 773, 774, 777, 778, 779, 759, 758, 757, 756, 755, 754, 753, 752, 751, 750, 748, 747, 746, 745, 744, 743, 742, 781, 782, 783, 806, 825, 824, 823, 821, 820, 818, 816, 815, 814, 813, 812, 811, 810, 808, 805, 784, 804, 803, 801, 799, 798, 796, 794, 793, 790, 789, 788, 787, 786, 785, 741, 739, 592, 654, 638, 640, 641, 642, 648, 651, 658, 738, 661, 664, 666, 668, 670, 671, 634, 631, 627, 624, 623, 620, 618, 616, 611, 610, 609, 608, 607, 605, 602, 597, 596, 672, 673, 674, 715, 737, 736, 734, 732, 730, 729, 728, 726, 724, 723, 722, 720, 718, 716, 714, 677, 712, 708, 706, 704, 703, 700, 699, 698, 697, 691, 690, 686, 685, 682, 557, 414, 555, 193, 218, 215, 213, 212, 210, 209, 208, 207, 206, 204, 198, 197, 196, 194, 192, 224, 191, 190, 189, 185, 184, 182, 179, 177, 176, 175, 174, 173, 172, 171, 221, 227, 169, 254, 273, 272, 271, 270, 269, 268, 265, 264, 262, 261, 260, 259, 258, 256, 253, 230, 252, 249, 248, 247, 245, 244, 243, 241, 240, 239, 237, 235, 234, 231, 170, 168, 551, 53, 87, 85, 82, 81, 80, 79, 75, 74, 70, 69, 62, 58, 57, 54, 47, 95, 46, 45, 40, 39, 36, 35, 30, 26, 24, 18, 13, 11, 8, 6, 92, 97, 167, 142, 166, 165, 164, 163, 161, 157, 155, 154, 152, 151, 149, 147, 146, 143, 138, 101, 133, 132, 131, 130, 128, 126, 122, 121, 120, 117, 114, 113, 111, 110, 274, 275, 276, 374, 399, 391, 387, 386, 385, 384, 383, 382, 381, 380, 379, 377, 376, 375, 373, 402, 372, 371, 370, 369, 367, 366, 365, 364, 363, 362, 361, 360, 358, 357, 400, 406, 277, 480, 549, 537, 534, 525, 523, 521, 519, 515, 512, 509, 506, 494, 490, 482, 478, 408, 469, 463, 459, 457, 453, 444, 439, 435, 434, 430, 425, 420, 2, 413, 356, 355, 354, 294, 315, 311, 310, 309, 307, 306, 305, 304, 303, 301, 299, 298, 297, 296, 293, 353, 292, 291, 290, 288, 287, 286, 285, 284, 283, 282, 281, 280, 279, 278, 316, 317, 318, 319, 352, 351, 349, 348, 347, 346, 345, 344, 343, 342, 341, 340, 339, 338, 337, 336, 335, 334, 333, 332, 330, 329, 328, 327, 325, 324, 323, 321, 320, 826	1
635, 397, 405, 404, 403, 419, 675, 401, 295, 593, 633, 452, 636, 625, 586, 451, 398, 300, 647, 407, 632, 582, 630, 692, 460, 577, 556, 628, 416, 687, 626, 678, 412, 684, 411, 410, 681, 409, 679, 302, 396, 392, 637, 394, 600, 322, 653, 652, 619, 643, 603, 378, 326, 585, 427, 428, 429, 612, 442, 368, 584, 614, 646, 359, 655, 598, 446, 393, 331, 390, 665, 447, 389, 663, 662, 312, 422, 388, 595, 314, 660, 639, 622, 659, 350, 629, 689, 695, 96, 791, 107, 104, 792, 99, 98, 532, 118, 93, 795, 90, 533, 797, 84, 116, 119, 78, 518, 517, 145, 775, 776, 141, 139, 135, 562, 565, 780, 564, 522, 563, 124, 800, 802, 461, 15, 817, 27, 819, 23, 17, 16, 14, 547, 822, 12, 10, 9, 7, 554, 548, 545, 72, 807, 538, 539, 67, 64, 61, 59, 55, 544, 809, 542, 51, 50, 49, 48, 771, 153, 769, 513, 472, 242, 717, 473, 719, 474, 236, 233, 229, 228, 226, 225, 222, 727, 220, 246, 711, 251, 466, 462, 576, 267, 266, 464, 263, 705, 710, 467, 257, 707, 255, 468, 709, 219, 479, 217, 762, 749, 501, 502, 504, 569, 508, 763, 187, 162, 160, 159, 158, 567, 156, 216, 496, 570, 491, 214, 731, 211, 483, 735, 205, 485, 203, 201, 200, 199, 486, 733	2
497, 178, 499, 476, 500, 702, 701, 645, 644, 441, 688, 693, 511, 581, 438, 437, 566, 516, 568, 455, 683, 144, 669, 725, 667, 574, 232, 475, 308, 721, 313, 481, 572, 450, 238, 657, 656, 181, 579, 195, 454, 571, 443, 492, 470, 676, 713, 575, 250, 186, 289, 680, 436, 136, 421, 65, 529, 530, 613, 531, 89, 604, 561, 535, 423, 601, 560, 599, 66, 52, 615, 395, 543, 44, 546, 38, 550, 590, 21, 552, 588, 415, 5, 3, 528, 696, 526, 134, 694, 432, 123, 524, 140, 617, 621, 527, 520	3
488, 125, 73, 202, 580, 76, 108, 487, 536, 20, 127, 498, 83, 431, 507, 650, 477, 424, 510, 484, 606, 68, 578, 148, 449, 137, 223, 594, 56, 150, 514, 433, 540, 42, 589, 183, 587, 558, 553	4
103, 426, 541, 445, 418, 503, 63, 505, 60, 489, 417, 458, 465, 33	5
34, 86, 471, 37, 88, 29, 493, 71, 25, 100, 115, 456, 188, 102, 448, 649, 19, 495	6
28, 105, 440, 129	7
4, 41, 180, 91	8
94	9
77, 112	10
22, 106	11
43, 109, 31	12
32	18

### 2.3 Feature generation

Feature generation is the process of transforming raw data into a set of features that represents the diverse attributes of the data, often for classification or regression tasks [60]. Feature quality is a significant factor in developing a machine-learning model [61]. Following the addition of group IDs to the host-pathogen pair, each protein amino acid sequences were encoded to generate a fixed-length feature vector using the Amino Acid Composition (AAC). The AAC algorithm computes the occurrence rate for each type of amino acid found in a specific protein sequence [26], [32], [62], [63]. The 20 naturally occurring amino acids are: “ACDEFGHIKLMNPQRSTVWY”, and their rate of occurrence is computed by dividing the number of amino acid type *x* (Nx) by the length of the protein sequence *N*, as shown in Eq. 1:

(1) $$f\left(x\right)=\;\frac{\mathrm{Nx}}{N}\;x=\{A,C,D,E,F,G,H,I,K,L,M,N,P,Q,R,S,T,V,W,Y\}$$

AAC serves as a fundamental yet robust feature for predicting PPIs. It can be applied independently or integrated with protein domain information to identify novel interactions and validate previously established ones [64]. Also, AAC is computationally simpler and requires fewer feature vectors as compared to other methods, such as k-mers, where the number of features grows exponentially with k, leading to high dimensionality and increased computational cost [65]. AAC was chosen as the primary feature representation method after comparing the performance of its features with other encoding techniques, including Moran Correlation, Conjoint Triad, Amino Acid Pairs (AAP), and kmers. AAC demonstrated superior performance with a significant time advantage over the other methods. Furthermore, a correlation matrix of the 40-dimensional feature vector was generated to illustrate the relationships among the 40 features derived from host and pathogen protein sequences. The detailed matrix is provided in Supplementary File S1.

As presented in the preceding sections, non-interacting and interacting protein profiles were retrieved from databases. These sequences were categorized into host and pathogen protein sequences. Now, using the Independent Protein feature (IPF) representation method, each protein in a host-pathogen pair undergoes a feature encoding process, which transforms the protein sequences into feature vectors [66], [67], [68]. Following the encoding, host and pathogen feature vectors were concatenated to form combined feature vectors representing the interactions between the pairs.

For instance, when the feature encoding method AAC was utilized in IPF, for each sequence in these host-pathogen pairs, the AAC encoding method translated each protein sequence into a feature vector of 20 dimensions, representing the composition of the 20 standard amino acids. For instance, when a host protein sequence *h1* and a pathogen protein sequence *p1* were processed. Applying the AAC encoding method to *h1* generates a 20-dimensional feature vector, *vh*. Similarly, applying the AAC encoding method to *p1* generated another 20-dimensional feature vector, *vp*. An illustration is provided in (2), (3), (4).

(2) $$\mathrm{vh}={\left[0.05,\;0.10,\;0.07,\;0.08,\;0.34,\;0.23,\;0.98,\;0.12,\;0.77,\;\ldots ,\;0.22\right]}_{20}$$

(3) $$\mathrm{vp}={\left[0.03,\;0.12,\;0.35,\;0.25,\;0,85,\;0.21,\;0.48,\;0.83,\;0.46,\;\ldots ,\;0.43\right]}_{20}$$

To represent the interaction between *h1* and *p1*, these feature vectors are concatenated, resulting in a 40-dimensional feature vector:

(4) $$\mathrm{vhp}={\left[0.05,\;0.10,\;0.07,\;0.08,\;\ldots ,0.22,\;0.03,\;0.12,\;0.35,\;\ldots ,0.43\right]}_{40}$$

**Fig. 2:**
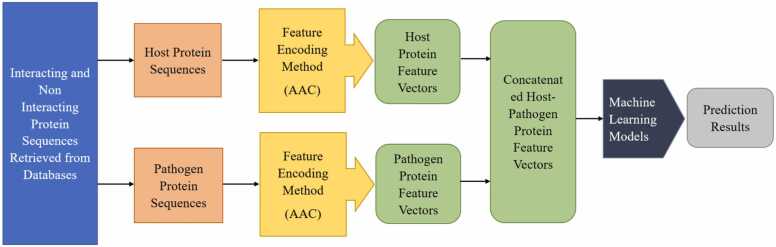
Feature generation using the Independent Protein Feature representation method.

Fig. 2 illustrates a workflow for converting protein sequences into feature vectors using AAC as the feature encoding method.

### 2.4 Modified differential evolution optimization method

Evolutionary algorithms are widely employed as global optimization techniques renowned for their resilience in handling noisy objective functions and their suitability for parallelization. These algorithms are rooted in biological evolution, replicating natural selection and continuously introducing new generations through recombination and mutation [69], [70]. New candidate solutions are generated through stochastic variations from existing parent individuals in each iteration or generation. Subsequently, individuals with higher fitness scores are selected as parents for the next generation, ensuring that each successive generation tends to produce individuals with improved fitness values [71], [72].

Traditional evolutionary optimization algorithms, such as Differential Evolution (DE), Genetic Algorithms (GA), and Evolution Strategies (ES), have shown varying levels of success in improving the efficiency and effectiveness of optimization processes [9], [73], [74]. A Genetic Algorithm (GA) is a search strategy inspired by Charles Darwin’s concept of natural selection. It involves five main phases: population initialization, fitness function evaluation, selection, crossover, and mutation [70], [73]. The process begins with a population of individuals, each represented by a finite-length vector analogous to a chromosome. The fitness function evaluates the competitiveness of each individual by assigning a performance-based score. This fitness score determines the probability of an individual being selected for the next generation. Selected individuals undergo crossover to produce offspring, followed by mutation to introduce variability [11], [75]. Evolutionary Strategies (ES) is another evolutionary algorithm rooted in natural selection principles. Unlike GA, ES does not employ crossover operations and relies exclusively on mutation to generate new solutions [72]. Differential Evolution (DE), maintains a population of candidate solutions represented as real-valued vectors, known as target vectors, each containing multiple decision variables [69], [76]. During each iteration, DE generates a donor vector through mutation and produces a trial vector via recombination. The best solution is selected using an approach that evaluates both target and trial vectors.

In the mutation phase, DE creates donor vectors by scaling the difference between two randomly selected vectors from the population and adding the result to a third vector. However, this approach can lead to redundant and suboptimal outcomes [11]. Therefore, the Modified DE introduces three key improvements. Firstly, the fitness scores of all vectors are calculated, and only the top three vectors are used to create donor vectors, reducing redundancy and avoiding suboptimal choices. Additionally, if the scaling factor is zero, the top-ranked vector is used as the donor vector instead of the first vector. Furthermore, when donor vector variables exceed boundaries due to scaling, an adaptive scaling factor is applied specifically to the out-of-bound variable. These modifications enhance the efficiency and effectiveness of DE, ensuring better exploration of the solution space and improved convergence. For contextual clarity, in DE optimization, vectors represent candidate solutions (or hyperparameter configurations) in the search space. The target vector is the current hyperparameter configuration under evaluation, while the donor vector is the new configuration generated through the processes of mutation and recombination.

The DE algorithm involves iterative processes of mutation, crossover, and selection to evolve a population of candidate solutions toward an optimal set of hyperparameters [69], [77], [78]. This study aims to leverage the strengths of DE by modifying its implementation to influence its generation of candidate solutions, thereby improving the selection of hyperparameter configurations using Expected Improvement. In the implementation of DE by [11] as shown in Algorithm 1, the donor vector is created using randomly generated indices from the population of target vectors. However, as an improvement, this study introduces a weighted approach for determining these indices, as opposed to the random method. In addition, the equation $V\leftarrow \lambda x_{1}+f*(\lambda x_{2}-\lambda x_{3})$ on line 17 was used to calculate the donor vector, where $x_{1},x_{2},x_{3}$ represent the random indices, $\lambda x_{i}$ represent the target vector at the ith index and f is the scaling factor. However, there were instances where the generated donor vector fell outside the specified hyperparameter bounds. Therefore, an adaptive approach for generating the donor vector is developed as a further contribution. Combining these two contributions, a new algorithm called Weighted and Adaptive Donor Vector (WADVec) was developed. Hence, both the process of generating the indices (line 16) and the computation of donor vectors (line 17) are modified in Algorithm 2.

Algorithm 1 Differential evolution algorithm [11]

**Table:** 

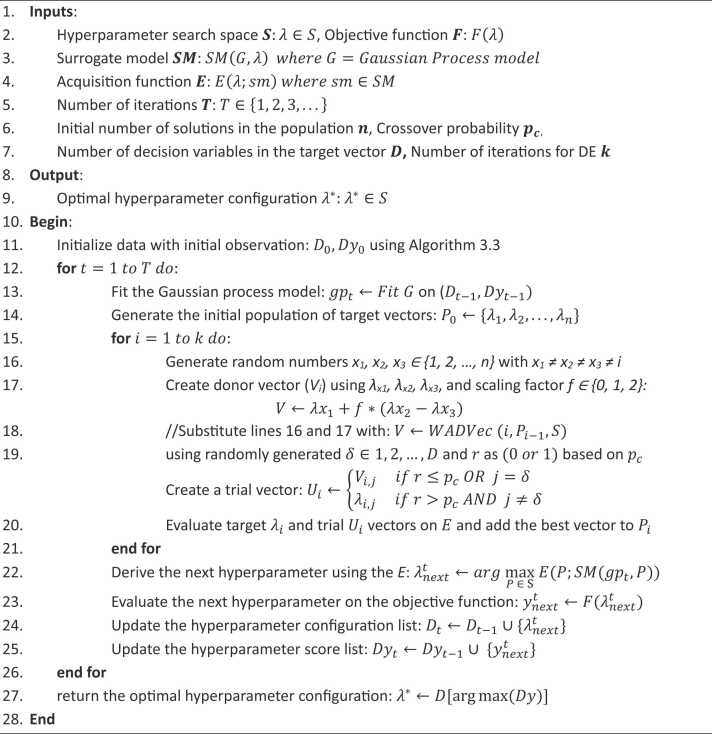

Algorithm 2 Weighted and adaptive donor vector (WADVec) algorithm

**Table:** 

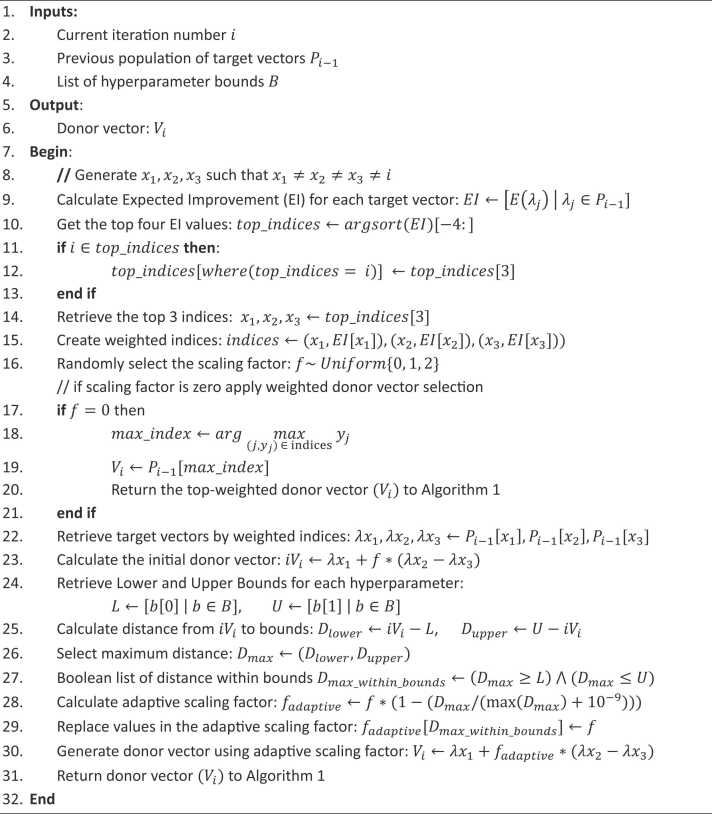

**Fig. 3:**
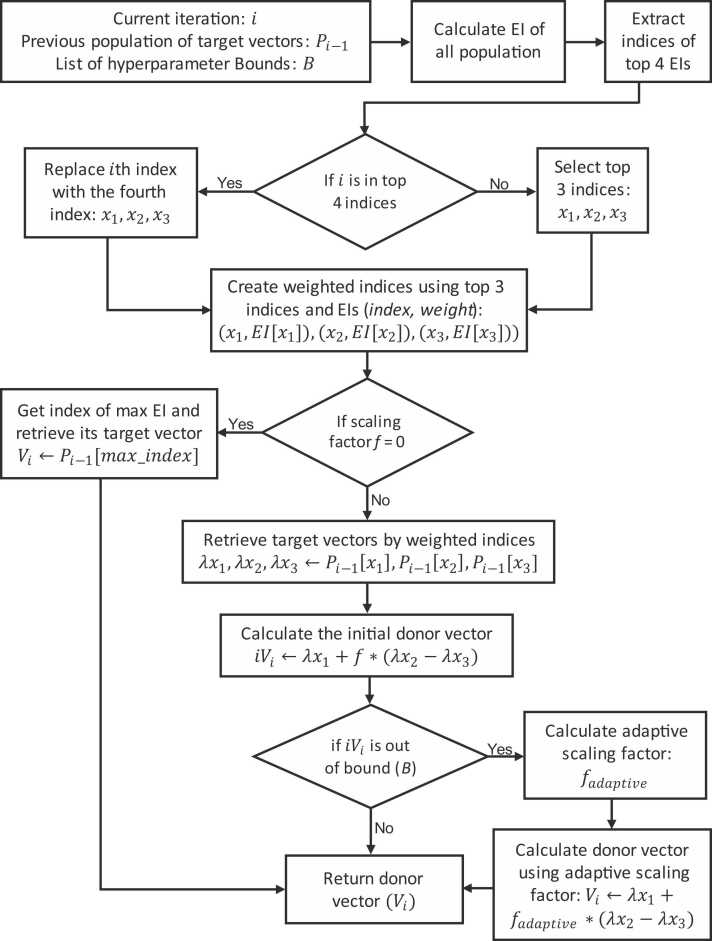
A workflow of the weighted and adaptive donor vector (WADVec) algorithm.

As shown in Algorithm 2 and Fig. 3, the WADVec procedure starts by calculating the Expected Improvement (EI) for each target vector in the previous population. In the traditional DE algorithm, the selected indices must be distinct and cannot coincide with the value of the current iteration number, $x_{1}\neq x_{2}\neq x_{3}\neq i$.

The indices of the top four target vectors based on EI values were identified. When the EI values are the same, the indices of the first four occurrences of the EI value are selected as a result of the sorting arrangement. If the current iteration number i is among the top three indices, it is replaced by the fourth-ranked index. The top three indices are then selected, and their corresponding EIs are used to serve as their weight, thereby creating weighted indices. Additionally, the algorithm identifies the index with the maximum EI when a scaling factor is chosen randomly from a uniform distribution of values {0, 1, 2} and the value is zero. It selects the corresponding vector from the previous population as the donor vector. If the scaling factor is not zero, the algorithm retrieves the target vectors corresponding to the weighted indices and calculates an initial donor vector using these target vectors.

The next step involves retrieving each hyperparameter's lower and upper bounds and calculating the distance from the initial donor vector to these bounds. The maximum distance is selected, and a Boolean list is created to identify distances within bounds. An adaptive scaling factor is then calculated, adjusted based on the distance to bounds, and used to generate the final donor vector. This adaptive scaling factor helps ensure that the donor vector remains within the defined hyperparameter bounds, which is only applied to the hyperparameter with an out-of-range donor vector value. The final donor vector is then returned to Algorithm 1 for further processing.

## 3 Results

A comprehensive suite of seven performance evaluation metrics was employed to evaluate and compare the predictive prowess of the models used in this study. These metrics encompassed standard measures such as Accuracy, Matthew’s Correlation Coefficient (MCC), recall (sensitivity), Area Under Receiver Operating Characteristic Curve (AUROC), specificity, F1 score, and precision. These metrics originated from a 2 × 2 matrix referred to as the confusion matrix [68]. They are the predominantly used metrics in classification tasks.

### 3.1 Evaluation of modified DE and other optimization methods

**Fig. 4:**
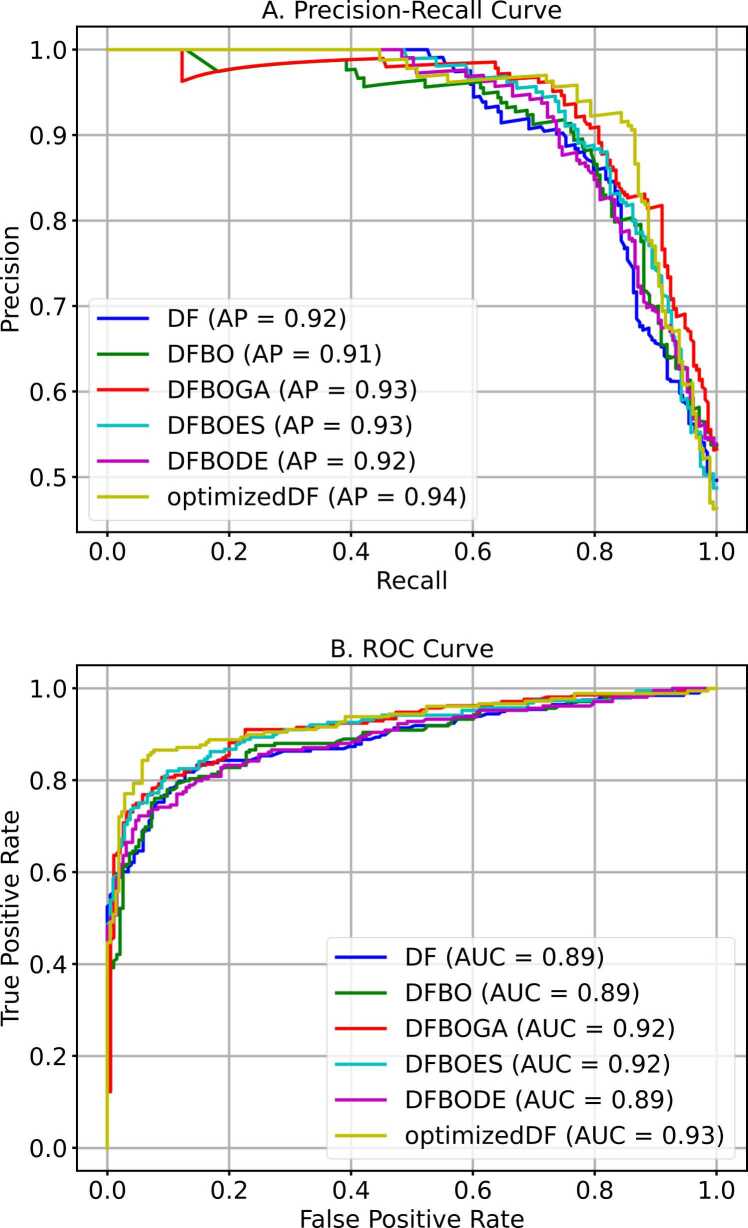
Hyperparameter optimization precision-recall and receiver operating characteristics curves. **A**. Precision-recall curve **B**. ROC curve.

**Fig. 5:**
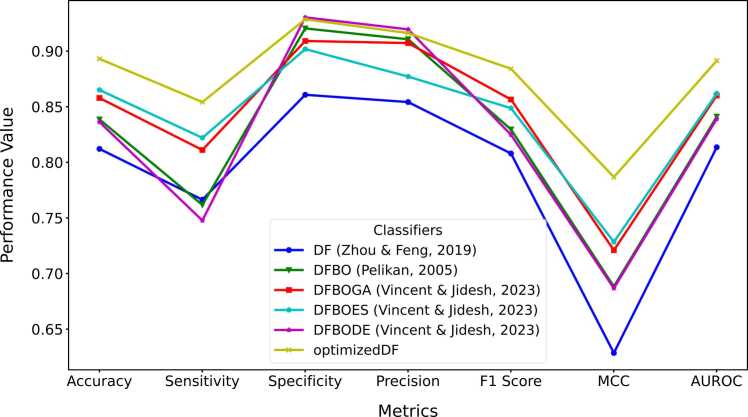
Hyperparameter optimization performance measures.

**Fig. 6:**
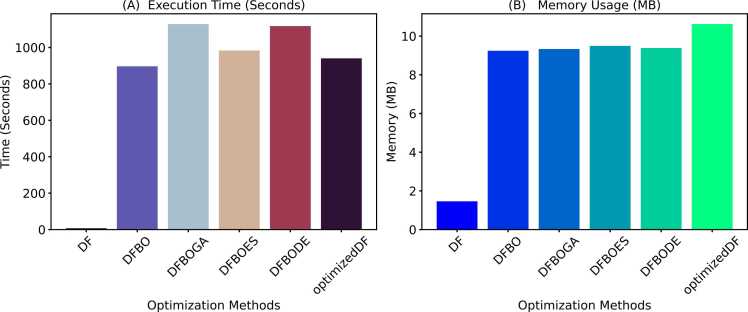
Comparison of execution time and memory usage across optimization methods. **A**. Execution time in seconds. **B**. Memory usage in megabytes (MB).

The central hypothesis of this study was that a competitive or better performance could be achieved by replacing the random selection of indices for target vectors used in computing donor vectors with a weighted and adaptive approach. The resulting modified DE was implemented in a deep forest model to evaluate the performance of the WADVec approach introduced in DE. Additionally, evolutionary algorithm optimization methods were implemented in deep forest for comparison. These methods included Bayesian Optimization (BO), Bayesian Optimization with Genetic Algorithm (BOGA), Bayesian Optimization with Evolutionary Strategies (BODE), and Bayesian Optimization with Differential Evolution (BODE). The goal of the comparison with standard traditional methods was to discover if the optimized DF (with modified DE) produced a competitive result compared to the aforementioned methods. Figs. 4A and 4B present the Precision-Recall and the receiver operating characteristics curves for each optimization method, respectively. Similarly, Fig. 5 provides more holistic performance results for the optimization methods, highlighting seven performance metrics. Additionally, the time and memory usage are also reported in Fig. 6.

### 3.2 Evaluation of optimized deep forest and traditional machine learning models

**Fig. 7:**
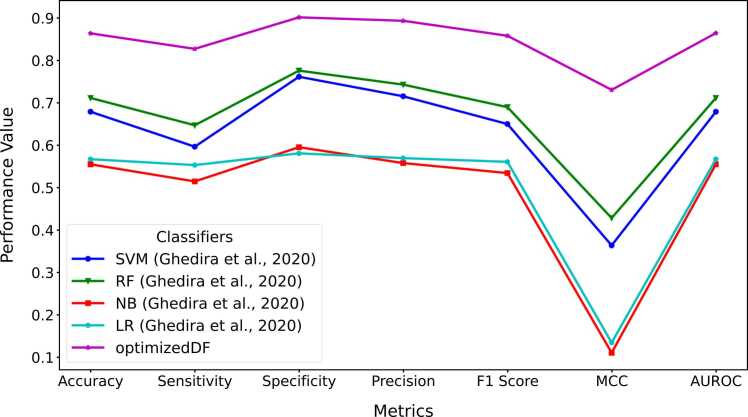
Performance comparison of various classifiers used by Ghedira *et al.*[79] and the optimized DF.

**Fig. 8:**
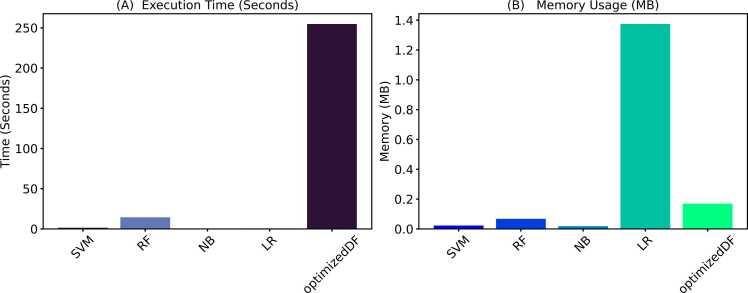
Comparison of execution time and memory usage with Ghedira *et al*. [79] machine learning models and the optimized DF. **A**. Execution time in seconds. **B**. Memory usage in megabytes (MB).

To rigorously evaluate the performance of the modified DE in the implemented optimized DF, this section presents and discusses results obtained through a comparison of the optimized deep forest model with traditional machine learning approaches. Additionally, it contrasts these findings with results from other optimization methods using a 10-fold cross-validation methodology. Fig. 7 presents a comparative analysis of various classifiers used by [79] in predicting human-*Plasmodium falciparum* interaction. Features were generated using the Term Frequency-Inverse Document Frequency (TD-IDF). The classifiers include Random Forest (RF), Support Vector Machine (SVM), Logistic Regression (LR), Naive Bayes (NB), and the optimized Decision Forest. Additionally, Fig. 8 provides an overview of each model's time and memory usage during the performance evaluation.

**Fig. 9:**
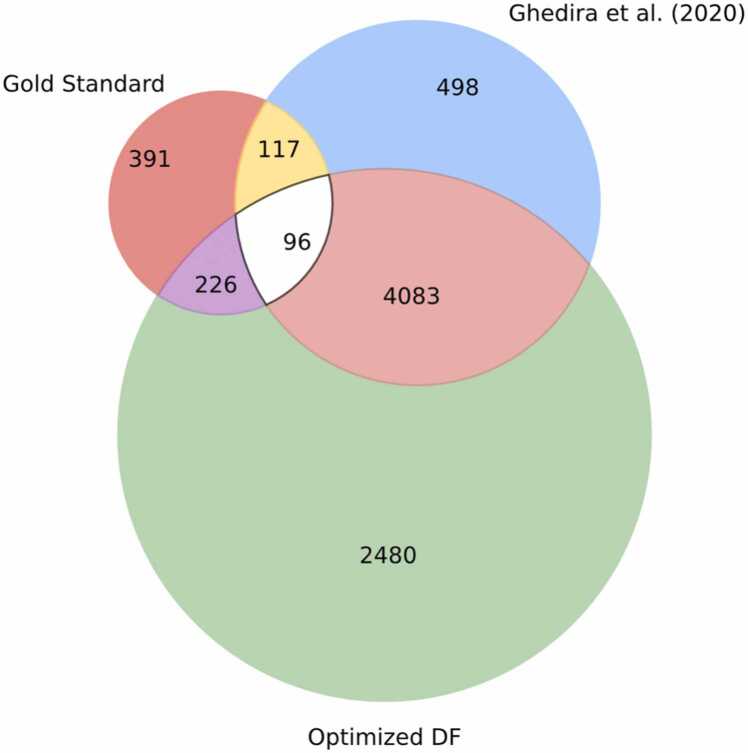
Venn diagram of gold standard HPPPI, Ghedira *et al*. [79] predicted HPPPI, and optimized DF predicted HPPPI.

For further validation of the optimized DF prediction model using human-*Plasmodium falciparum* (PF) PPI, labeled data containing 3000 interacting and 3000 non-interacting PPI were employed. The unlabeled data contains 8745 human-PF PPI samples. In total, 6885 of the 8745 were predicted as interacting proteins using the optimized DF-based prediction model. The prediction was validated using the 830 prepared gold standard interacting protein pairs discussed in Section 2.2. A total of 322 interacting partners, representing 38.8 %, were validated by the list of interacting pairs in the gold standard. Similarly, to evaluate the result from the optimized DF technique, the human-PF PPI dataset was predicted using the machine learning models used by [79] to predict human-PF interactions. For comparison, results from random forest, the top-performing model of [79], with a predicted total of 4794 PPIs as interacting pairs, were employed in this analysis. By comparing the optimized DF technique result to the results from [79], 4179 PPIs, representing 87.2 %, were commonly predicted as interacting proteins. Fig. 9 shows the intersection of the prediction from the optimized DF method, [79] and the gold standard.

### 3.3 Evaluation of optimized deep forest, decision trees, and neural network models

**Fig. 10:**
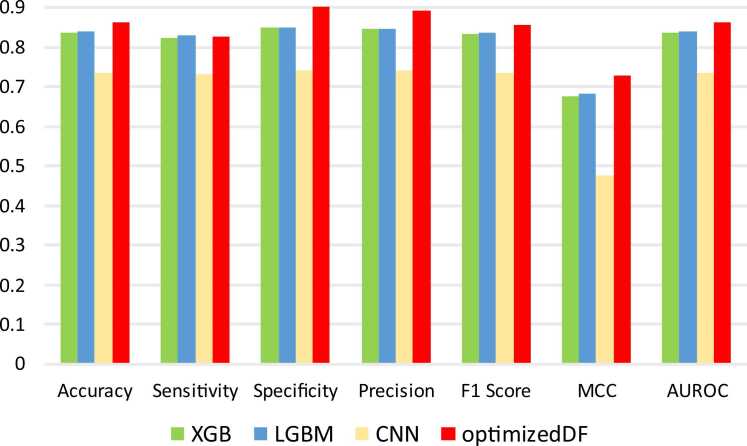
Performance comparison of eXtreme Gradient Boosting (XGB), Light Gradient Boosting Machine (LGBM), Convolutional Neural Network (CNN), and the optimized Deep Forest (optimized DF).

**Fig. 11:**
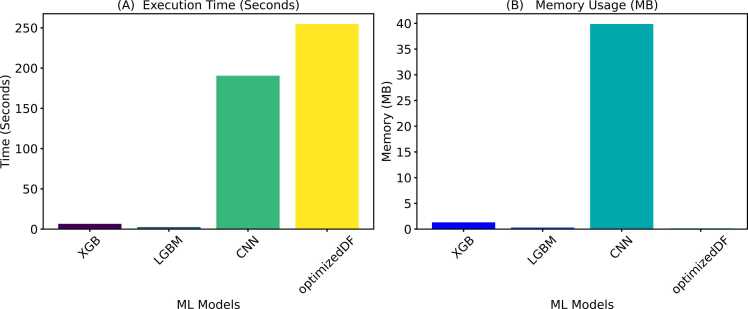
Comparison of execution time and memory usage using eXtreme Gradient boosting (XGB), Light gradient boosting machine (LGBM), Convolutional neural network (CNN), and the optimized deep forest (optimized DF). **A**. Execution time in seconds. **B**. Memory usage in megabytes (MB).

Furthermore, the performance of the optimized DF model developed in this research was compared with the performance of additional decision tree models and a neural network model. These models include eXtreme Gradient Boosting (XGBoost or XGB), Light Gradient Boosting Machine (LightGBM or LGBM), and Convolutional Neural Network (CNN). XGBoost, a gradient-boosting framework, is known for its efficiency and accuracy, making it a popular choice for many predictive modeling problems. LightGBM, another gradient boosting model, is designed for speed and efficiency, handling large datasets and high-dimensional data easily while often providing slightly improved performance over XGB. CNNs are primarily used in image processing and pattern recognition, and they leverage their ability to learn spatial hierarchies of features automatically. However, they may not always excel in traditional tabular data tasks. Fig. 10 shows these models’ evaluation performance metrics through 10-fold cross-validation on the human-*Plasmodium falciparum* dataset. Also, Fig. 11 provides an overview of each model’s time and memory usage during the performance evaluation.

### 3.4 Evaluation of optimized deep forest on independent dataset

Furthermore, following the training of the optimized deep forest model, a comprehensive validation process was initiated using a dataset comprising previously predicted non-interacting host-pathogen interactions. This dataset, originally extracted from the study conducted by Agamah et al. [79], was selected to test the model's predictive capabilities. The dataset contained 4982 valid records after preprocessing, each representing a pair of host and pathogen proteins previously classified as non-interacting using network prediction.

Upon processing this dataset, the system utilized the optimized deep forest model to re-evaluate the interactions. Remarkably, out of the 4982 records, the model identified 1239 interactions as potential positive interactions and 3743 as negative interactions. This result suggests a significant advancement in the proposed system's ability to predict host-pathogen interactions, as these pairs were previously not recognized as non-interacting. Table 3 lists the top 10 of the predicted human-*Plasmodium falciparum* interacting proteins, along with their annotations. Each entry in the table includes the protein identifiers, functional descriptions, and relevant biological annotations that support the predicted interactions. Also, the first 3 host-pathogen PPI in Table 2, such as P50250 - P08319, Q8ILI6 - O94813, and Q7KQL3 - Q96GQ7, have been reported in the literature, while the remaining seven host-pathogen PPI are the novel interactions predicted by our model (in red color). The biological relevance of these interactions is presented in the discussion section (Section 4.0).

**Table 3:** Predicted Human-*Plasmodium falciparum* (PF) protein-protein interaction.

Pathogen ID	Annotation	Host ID	Annotation
P50250	Adenosylhomocysteine	P08319	All-trans-retinol dehydrogenase ADH4.
Q8ILI6	Acidic leucine-rich nuclear phosphoprotein 32-related protein	O94813	Slit homolog 2 protein. Thought to act as a molecular guidance cue in cellular migration
Q7KQL3	ADP-ribosylation factor 1.	Q96GQ7	Probable ATP-dependent RNA helicase DDX27.
Q8I4X0	Actin 1. A highly conserved protein that polymerizes to produce filaments.	O96019	Actin-like protein 6 A. Involved in transcriptional activation and repression of select genes.
Q8I4X0	Actin 1. A highly conserved protein that polymerizes to produce filaments.	P61160	Actin-related protein 2. ATP-binding component of the Arp2/3 complex
C0H4W3	Probable ATP-dependent helicase PF08_0048.	Q9NS87	Kinesin-like protein KIF15. Plus-end directed kinesin-like motor enzyme involved in mitotic spindle assembly.
Q8I1T8	ATPase ASNA1 homolog. ATPase required for the post-translational delivery of tail-anchored (TA) proteins.	Q9UKX3	Myosin−13. Fast-twitching myosin mediates the high-velocity and low-tension contractions of specific striated muscles.
P46468	Putative cell division cycle ATPase.	Q9Y4C4	Malignant fibrous histiocytoma-amplified sequence 1. Probable GTP-binding protein.
Q8IDR3	Myosin-A. Myosins are actin-based motor molecules with ATPase activity.	P55039	Developmentally-regulated GTP-binding protein 2.
Q8ILT5	Protein SEY1 homolog. Probable GTP-binding protein	Q8TAI7	GTPase RhebL1. Binds GTP and exhibits intrinsic GTPase activity.

To provide a more comprehensive validation of the predictive performance of the optimized Deep Forest (DF) model, evaluations were further conducted on two distinct datasets, these include a customer churn dataset and the Modified National Institute of Standards and Technology (MNIST) image dataset, both retrieved from Kaggle. The primary aim of choosing these two datasets is to evaluate the model's performance on non-protein sequence datasets, specifically image and categorical datasets.

The bank customer churn dataset includes information about a bank's customers, with the target variable being a binary variable that shows whether a customer has left the bank (closed their account) or remains a customer. The extracted dataset used in this evaluation consists of 4074 records with 14 features, namely, row number, surname, customer ID, geography, credit score, age, gender, tenure, balance, number of bank products the customer is using, estimated salary, is-active member, has-credit-card and the target variable (Exited). For this analysis, row number and customer ID were removed as they are of no importance to this evaluation. Hence only 12 features were eventually used. The MNIST dataset is a subset derived from a larger dataset provided by NIST. The original black and white images from NIST were size normalized to fit in a 20 × 20 pixel box while preserving their aspect ratio. The resulting images contain grey levels as a result of the anti-aliasing technique used by the normalization algorithm. The dataset contains 60,000 images of handwritten digits across 10 classes, each class representing each of the 10 digits. The digits have been size-normalized and centered in a fixed-size image. Furthermore, for the purpose of this evaluation and to ensure binary classification, two classes of images were employed. These categories produced a total of 11,000 image samples.

The customer churn dataset assesses the model’s ability to predict whether customers are likely to leave a service, while the MNIST dataset evaluates its performance in image classification tasks involving handwritten digits. In both experiments, the DF model optimized using the modified Differential Evolution (DE) algorithm demonstrated better performance than the traditional DE, and it presented competitive results compared to the model optimized with alternative techniques. The detailed performance results, including accuracy, precision, recall, and F1 score, are presented in the supplementary file S1.

### 3.5 Implementation of optimized deep forest as a web application

**Fig. 12:**
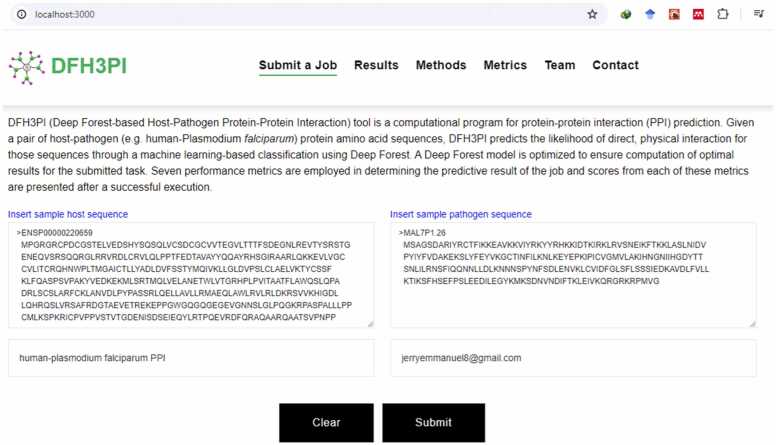
Interface of the DFH3PI webserver for HPPPI prediction.

**Fig. 13:**
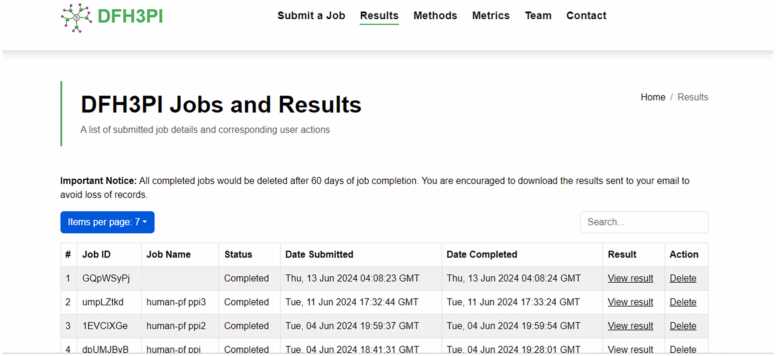
DFH3PI results page displaying list of submitted jobs.

The optimized model was implemented as a web prediction tool to solve the problems of host-pathogen protein-protein interaction prediction where limited data are available. The web application, named DFH3PI (Deep Forest Host-Pathogen Protein-Protein Interaction), is illustrated in Fig. 11, Fig. 12, Fig. 13 and accessible at https://dfh3pi.covenantuniversity.edu.ng. The web application's front-end was developed using React, a JavaScript framework. The frontend consists of a job submission page, a result page, and other important pages. The backend application that handles requests from the front-end was developed using Flask, a Python programming language framework. A database that stores submitted information and the corresponding results produced was developed using PostgreSQL. An NGINX server was configured to serve the frontend application and as a proxy for routing requests to the backend application program interface (API) routes. A web server gateway interface (WSGI) was also configured to serve the backend application and to keep the application running.

In Fig. 12, the web interface allows users to input a pair of host-pathogen protein sequences for analysis. The text boxes can be pre-populated with sample sequences to guide users by clicking on the “*Insert sample host sequence”* or the “*Insert sample pathogen sequence”*. Users can also name their jobs and provide an email address to receive results. Upon submission, the web server processes the sequences using the optimized DF model and returns predictions on the likelihood of interaction between the proteins.

This webserver leverages the optimized DF model's superior performance metrics, including its high accuracy, sensitivity, specificity, precision, F1 score, MCC, and AUROC, to ensure reliable predictions. Despite the computational demands typically associated with machine learning models, the optimized DF model maintains efficient execution time and memory usage, making it suitable for deployment in a user-facing web application.

**Fig. 14:**
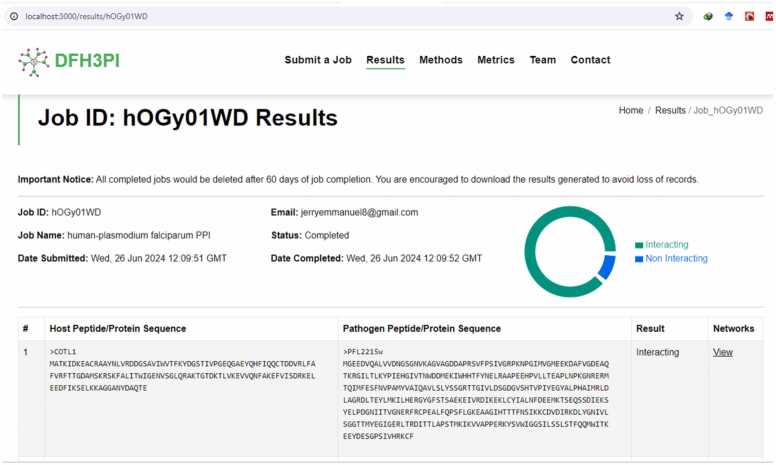
DFH3PI job result page displaying prediction results and link to view networks.

Fig. 13 displays the list of results on the DFH3PI webserver. Users must provide an email address to view or delete a completed or submitted result, ensuring a straightforward yet effective privacy mechanism for submitted tasks. This approach helps maintain the confidentiality of user data and results. It is important to note that jobs in progress cannot be deleted, preserving the integrity of ongoing analyses. Once a job is completed, users can access detailed information about the job and the interaction results, as depicted in Fig. 14.

## 4 Discussion

The comparison of performance metrics among different optimization methods, as displayed in Fig. 5, Fig. 6, highlights the substantial advancements achieved through hyperparameter optimization, particularly evident in the performance of the modified DE-based DF. Starting with the baseline DF model, which attained an accuracy of 81.20 %, subsequent optimization techniques such as DFBO (83.86 %), DFBOGA (85.78 %), DFBOES (86.51 %), and DFBODE (83.61 %) progressively improve across various metrics including sensitivity, specificity, F1 score, precision, AUROC and Matthew’s correlation coefficient (MCC). However, amongst these methods, DF with the modified DE (optimized DF) emerged as the top performer with an impressive accuracy of 89.30 %. This model not only surpassed the baseline and other optimization methods but also exhibited notable enhancements in sensitivity (85.42 %), specificity (92.86 %), precision (91.62 %), F1 score (88.41 %), MCC (78.67 %), and AUROC (89.14 %).

As shown in Figs. 6A and 6B, despite achieving high accuracy, the optimized DF model demonstrates exceptional efficiency in terms of time and memory utilization compared to other optimization methods. Specifically, optimized DF completed its training and prediction tasks in 940 seconds, which, although higher than the baseline DF model, is significantly lower than the traditional DE and remains competitive considering the complex optimization process involved. Moreover, optimized DF utilized 10.62 MB of memory, slightly higher than other optimized methods but still demonstrating efficient resource management.

In the performance evaluation of deep forest and traditional machine learning models, as depicted in Fig. 7, the Support Vector Machine (SVM) exhibits moderate performance across the metrics. It achieves an accuracy of 0.679, which indicates that around 68 % of the predictions are correct. The sensitivity, which measures the true positive rate, is 0.596, implying that the model correctly identifies approximately 60 % of the positive interactions. The specificity, at 0.761, is higher, suggesting that the model is better at identifying non-interactions. The precision, or the proportion of true positive predictions, is 0.715. The F1 score, which balances precision and sensitivity, stands at 0.650. However, the MCC of 0.363 and AUROC of 0.679 show room for improvement in capturing the true predictive power of the model. Random Forest (RF) performs better than SVM with an accuracy of 0.711. Its sensitivity is 0.647, indicating a better true positive rate than SVM. The specificity is 0.776, demonstrating a strong ability to identify non-interactions. RF's precision is 0.743, and its F1 score is 0.690, both higher than SVM, reflecting a better balance between recall and precision. The MCC of 0.428 and AUROC of 0.711 confirm its improved performance over SVM.

Naive Bayes (NB) shows the weakest performance among the models, with an accuracy of 0.555. Its sensitivity is 0.514, meaning it correctly identifies just over half of the positive interactions. The specificity is 0.595, indicating a lower ability to identify non-interactions. The precision is 0.558, and the F1 score is 0.534, both suggesting limited effectiveness in balancing precision and recall. The MCC is very low at 0.110, and the AUROC is also the lowest at 0.555, highlighting its overall poor performance. Logistic Regression (LR) performs slightly better than NB, with an accuracy of 0.567. Its sensitivity is 0.553, showing a marginally better true positive rate. The specificity is 0.581, indicating a limited ability to identify non-interactions. Precision is 0.569, and the F1 score is 0.561, which are improvements over NB but still not high. The MCC of 0.134 and AUROC of 0.567 reflect the model's modest performance.

The optimized Decision Forest significantly outperforms all the other classifiers. It achieves an impressive accuracy of 0.864, indicating that over 86.4 % of the predictions are correct. The sensitivity is 0.827, showing a high true positive rate, and the specificity is 0.902, highlighting its excellent ability to identify non-interactions. The precision is 89.4 %, and the F1 score is 85.8 %, demonstrating a superior balance between precision and recall. The AUROC of 0.865 further confirms the model's ability to discriminate between positive and negative interactions. Compared to the models used by [79], the optimized DF model shows a substantial increase in performance across all metrics. This significant improvement, despite using a 10-fold cross-validation, underscores the effectiveness of the optimized Decision Forest approach in predicting host-pathogen protein-protein interactions.

Similarly, in the performance evaluation of deep forest with XGB, LGBM, and CNN in Fig. 10, the optimized DF model demonstrates superior performance across all key metrics compared to XGBoost (XGB), LightGBM (LGBM), and Convolutional Neural Network (CNN). It achieves the highest accuracy at 0.864, significantly outperforming XGB (0.837) and LGBM (0.840), and well above the CNN (0.737). In terms of sensitivity, which indicates the model’s ability to correctly identify positive instances, optimized DF maintains competitive performance at 0.827, closely trailing LGBM (0.830) but surpassing XGB (0.825) and the CNN (0.733). Its specificity is notably high at 0.902, reflecting its strong performance in correctly identifying negative instances, a considerable improvement over XGB (0.849), LGBM (0.851), and far superior to CNN (0.741). The optimized DF also excels in precision with a value of 0.894, surpassing XGB (0.848) and LGBM (0.848), and achieves a commendable F1 Score of 0.858, outperforming XGB (0.835) and LGBM (0.838), and significantly surpassing CNN (0.735). Furthermore, the Matthews Correlation Coefficient (MCC) and AUROC scores for optimized DF (0.730 and 0.864, respectively) indicate a better overall balance between classification metrics compared to the other models, with XGB and LGBM scores of 0.676 and 0.682 for MCC and 0.837 and 0.840 for AUROC respectively, and a notable gap compared to CNN's lower scores. Thus, the optimized DF model is the most robust and reliable classifier in this comparison.

Furthermore, the optimized DF model was evaluated on the human–SARS-CoV-2 (virus) dataset, where it outperformed existing methods across most metrics, including accuracy, sensitivity, and F1 score. The results of this evaluation are presented in the supplementary file.

With regard to the results obtained on the independent dataset, the three predicted host-pathogen PPIs which have been reported in the literature are discussed. Discussions on the seven novel interactions are provided in the supplementary file S1 Table 1. The first predicted interaction involves the human protein All-trans-retinol dehydrogenase ADH4 (P08319) and the *Plasmodium falciparum* protein Adenosylhomocysteine (P50250). Adenosylhomocysteine acts as a competitive inhibitor of S-adenosyl-L-methionine-dependent methyl transferase reactions and plays a crucial role in regulating intracellular concentrations of adenosylhomocysteine [54], [80]. On the other hand, ADH4 catalyzes the Nicotinamide Adenine Dinucleotide (NAD)-dependent oxidation of either all-trans-retinol or 9-cis-retinol [81]. Sanchez-Rocha et al. [82] reported that these proteins are part of 1393 UniProt codes in the same sequence identity cluster, indicating that they could interact based on UniProt codes of Protein Data Bank in Europe (PDBe) entries with a 90 % sequence identity cluster. Agamah *et al*. [55] also noted some percentage of sequence similarity between these proteins.

Similarly, an interaction is predicted between the human protein Slit homolog 2 (O94813) and the *Plasmodium falciparum* protein Acidic leucine-rich nuclear phosphoprotein 32-related protein (Q8ILI6). SLIT2 functions as a molecular guidance cue in cellular migration, mediated by its interactions with roundabout homolog receptors, as described by [83]. Initially, Q8ILI6 was predicted by [51] to interact with the apurinic/apyrimidinic endodeoxyribonuclease APEX1 (P27695). APEX1 is a multifunctional protein that plays a central role in the cellular response to oxidative stress. The two major activities of APEX1 are DNA repair and redox regulation of transcriptional factors. However, further research revealed that Q8ILI6 also interacts with SLIT2. This interaction is further substantiated by evidence showing that SLIT2 has a direct physical high-throughput interaction with APEX1, as documented by [84].

The third predicted interaction features the human protein Probable adenosine triphosphate (ATP)-dependent RNA helicase Dead-box 27 (DDX27) (Q96GQ7) and the *Plasmodium falciparum* protein Adenosine Diphosphate (ADP)-ribosylation factor 1 (Q7KQL3). DDX27 plays a critical role in the nucleolar ribosomal RNA processing machinery, specifically regulating the 3′ end formation of ribosomal 47S rRNA, a crucial step in ribosome biogenesis [85]. This protein is essential for the proper assembly and function of ribosomes, which are necessary for protein synthesis in human cells. On the other hand, Q7KQL3, a small guanosine triphosphate (GTPase), is integral to protein trafficking between different cellular compartments, facilitating the movement and sorting of proteins within the cell [86]. GTPases like Q7KQL3 act as molecular switches that control various cellular processes by cycling between active (GTP-bound) and inactive (guanosine diphosphate (GDP)-bound) states. According to [87], DEAD-box helicases such as DDX27 typically exhibit a higher affinity for binding ADP-ribosylation factors like Q7KQL3. This affinity suggests that DDX27 may interact with Q7KQL3 to coordinate or regulate processes involving ribosome biogenesis and protein trafficking.

To evaluate the DFH3PI web application, a questionnaire covering the application’s usability on different devices, result presentation, error handling, predictive accuracy, response time, and data security was administered to bioinformaticians and computational biologists. Additionally, DFH3PI was compared with Protein Prompt in terms of innovative features. Based on the responses received, 100 % of respondents opined that DFH3PI offers competitive innovative features compared to Protein Prompt. Other results indicate high satisfaction in usability, with 80 % of respondents being very satisfied and 20 % satisfied. Compatibility of the application on different devices and presentation of the results generated show a split, with 40 % being very satisfied and 60 % satisfied in each category. Error handling and innovation have perfect scores, with 100 % of respondents being very satisfied and none expressing dissatisfaction. Similarly, crashes or errors reveal that 80 % of the respondents reported no errors, while 20 % experienced some network errors. These errors were a result of the general network downtime in the University as the application is hosted on the University network infrastructure. However, this has since been resolved. Furthermore, the Accuracy and data security are well-regarded, with 80 % of respondents very satisfied with the prediction accuracy of the application, though 20 % are neutral. All respondents were satisfied with the application's performance (input, actions, and response time). Overall satisfaction sees all respondents split between very satisfied and satisfied.

Despite the enhanced predictive performance of the model developed in this study, future work would explore incorporating structural features such as binding pockets and protein folds in predicting protein-protein interactions. Integrating these features could enhance both the accuracy and interpretability of the optimized model by offering deeper insights into the predicted interactions. In addition, while the optimized DF model has demonstrated improved performance across various datasets, including other PPI datasets, its web application (DFH3PI) was implemented exclusively for human-*Plasmodium falciparum* PPIs to ensure strict compliance with the objectives set by the funders of this study. Future work will aim to scale the application to support multi-organism PPI prediction. Also, the sequences employed in this study were retrieved from the literature as used by other researchers. No model or human samples were directly involved in this study; hence, it is recommended that the developed approach be further evaluated for broader sample types using samples from diverse biological sources, including both model organisms (animal, cellular) and human tissues (biopsies, postmortem) to assess its generalizability and performance.

## 5 Conclusion

In this study, a modified differential evolution (DE) optimization method was developed to improve candidate hyperparameter configuration selection. This modified DE approach was evaluated and compared with existing and state-of-the-art evolutionary algorithms, including genetic algorithms, evolutionary strategies, standard differential evolution, and Bayesian optimization. The results showed that the modified DE outperformed these methods across standard classification-based performance metrics such as accuracy, sensitivity, specificity, precision, F1 score, Matthew's correlation coefficient (MCC), and area under the ROC curve (AUROC). Additionally, the modified DE demonstrated competitive time and memory usage in several experiments. The modified DE was then implemented for automatic hyperparameter selection in the deep forest model, creating an optimized deep forest (DF) model. This optimized DF model outperformed traditional models such as random forest, support vector machine (SVM), gradient boosting machine (GBM), and convolutional neural network (CNN) in predicting human-*Plasmodium falciparum* protein-protein interactions. To further validate the predictive capabilities of the optimized DF model, it was evaluated using a customer churn dataset and the Modified National Institute of Standards and Technology (MNIST) image dataset. In both cases, the optimized DF model (using modified DE) produced competitive results compared to the DF model optimized with other optimization methods. The detailed results of these experiments are provided in the supplementary materials of this study. Finally, the optimized DF model was employed to identify potential interacting protein partners and was implemented as a web tool for predicting protein-protein interactions.

## CRediT authorship contribution statement

**Emmanuel Jerry:** Writing – review & editing, Writing – original draft, Visualization, Validation, Software, Methodology. **Isewon Itunuoluwa:** Writing – review & editing, Writing – original draft, Visualization, Validation, Supervision, Software, Resources, Project administration, Methodology, Funding acquisition, Formal analysis, Data curation, Conceptualization. **Oyelade Jelili:** Writing – review & editing, Writing – original draft, Visualization, Validation, Supervision, Software, Resources, Project administration, Methodology, Funding acquisition, Formal analysis, Data curation, Conceptualization.

## Declaration of Competing Interest

The authors declare that they have no known competing financial interests or personal relationships that could have appeared to influence the work reported in this paper.

## Data Availability

All the datasets used in this work are publicly available on GitHub and accessible at https://github.com/JerrySteam/Optimized-DF-Using-WADVec
