# Supplementary material for: An optimized deep-forest algorithm using a modified differential evolution optimization algorithm: A case of host-pathogen protein-protein interaction prediction
Source: Comput Struct Biotechnol J. 2025 Jan 26;27:595–611. doi: 10.1016/j.csbj.2025.01.020 (PMC11849198; doi:10.1016/j.csbj.2025.01.020)
Supplement: Supplementary file 1 — Supplementary material [file mmc1.docx]

**A. Amino Acid Composition Correlation Matrix**

A key observation to note from the matrices is that no two features exhibited a strong negative or positive correlation to each other, thereby implying the absence of multicollinearity amongst the features. This further strengthens the resolve to employ the use of all the features in the developed HPPPI model.


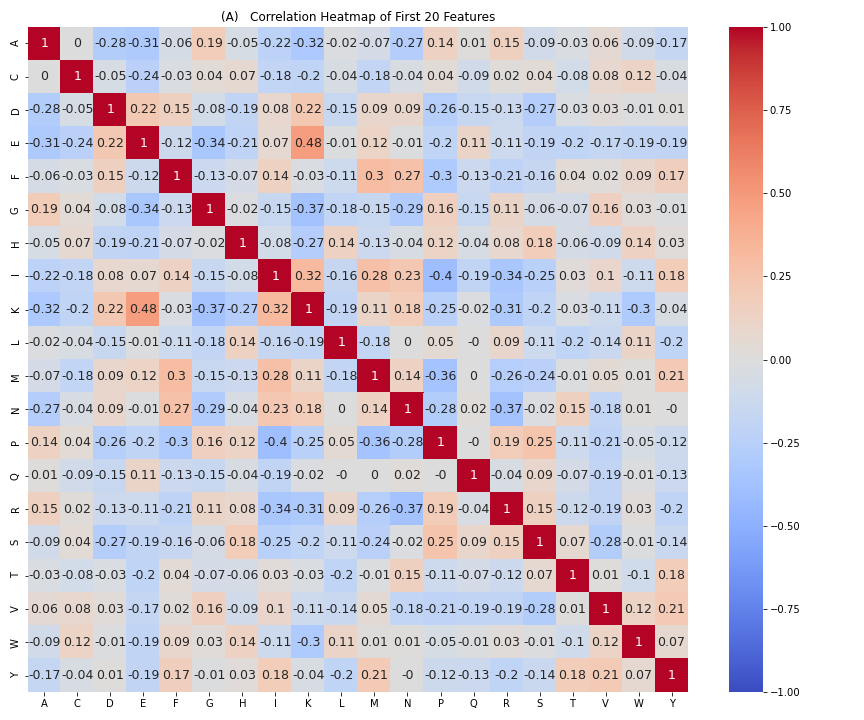


**Figure 1:** Correlation matrix of host amino acid composition features


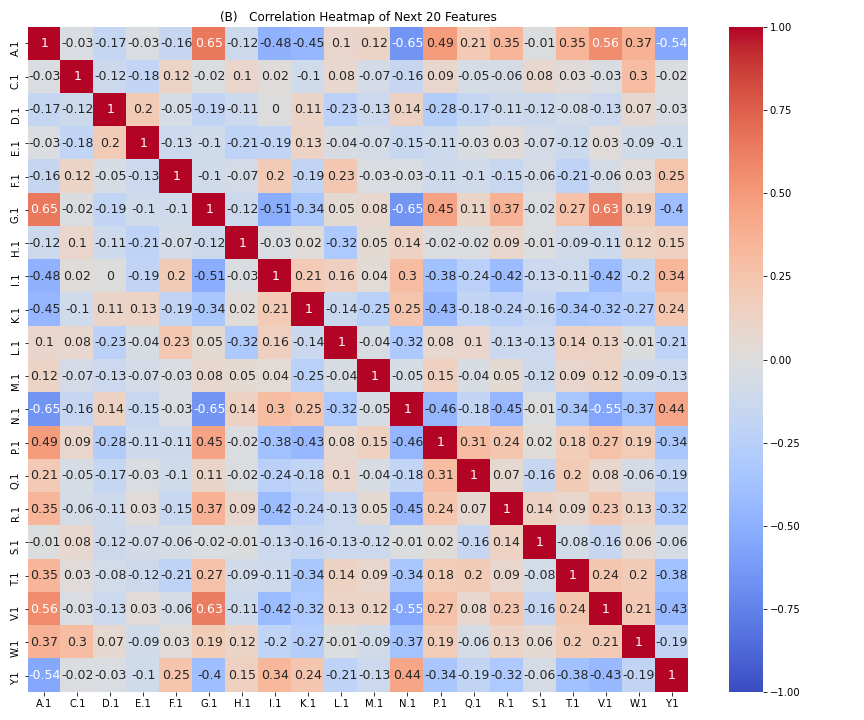


**Figure 2:** Correlation matrix of pathogen amino acid composition features

**B. Protein Sequence Data Grouping**

Given the nature of HPPPI sequences, random partitioning of interacting pairs during cross-validation or train-test splitting can lead to inconsistent prediction results. For instance, if a host protein interacts with multiple pathogen proteins, random splitting can lead to an overlap of the same host across multiple folds, resulting in inconsistency in model training and testing. To address this issue, a group-splitting approach was implemented. This method forms folds or splits by considering dataset groups and aims to maintain the sample distribution across classes within each fold as much as possible while ensuring that groups do not overlap between folds. During data preparation, a method was developed to group interacting pathogen and host protein pairs according to the similarity of the amino acid sequences of the host proteins. The groups and their corresponding group IDs were generated using the steps outlined in the algorithm below.

**Algorithm:** Generate Protein Sequence Group IDs

1. **Inputs**:
2. Series of protein amino acids: $sequences$
3. **Output**:
4. Group IDs: $group_{ids}$
5. **Begin**:
6. Initialize: Set $G\leftarrow0$ and $group_{ids}\leftarrow0$
7. **for** each $(n_{i}, s_{i})$ in $sequences:$ // Group by Sequence
8. ${G(s}_{i}) \leftarrow{G(s}_{i})\cup\{n_{i}\}$
9. **end for**
10. **for** each unique $s_{j}$ in $G$: // Assign group IDs
11. $g_{j}\leftarrow g_{0}+j-1$
12. **for** each $n_{i}$ associated with $s_{j}$:
13. $group_{ids}[n_{i}]\leftarrow g_{j}$
14. $group_{ids}\leftarrow group_{ids} \cup group_{ids}\left[ n_{i} \right]$
15. **end for**
16. **end for**
17. return $group_{ids}$

Across all training datasets, comprising 1,660 non-interacting and interacting pairs, 834 group IDs were derived. Consequently, rather than randomly splitting the pathogen and host pairs, the group-splitting approach guarantees that any interacting host-pathogen pair sharing identical group ID is not included in both the training fold and validation fold simultaneously during cross-validation or train-test splitting. This method ensures that a host protein, which interacts with various pathogen proteins but has the same group ID, does not appear in both the training and testing sets.

**C.** Predicted Human-*Plasmodium falciparum* (PF) protein-protein interaction

**Table:** Predicted Human-*Plasmodium falciparum* (PF) protein-protein interaction

| Pathogen Uniprot ID | Annotation | Host Uniprot ID | Annotation |
| --- | --- | --- | --- |
| P50250 | Adenosylhomocysteine | P08319 | All-trans-retinol dehydrogenase ADH4. |
| Q8ILI6 | Acidic leucine-rich nuclear phosphoprotein 32-related protein | O94813 | Slit homolog 2 protein. Thought to act as a molecular guidance cue in cellular migration |
| Q7KQL3 | ADP-ribosylation factor 1. | Q96GQ7 | Probable ATP-dependent RNA helicase DDX27. |
| Q8I4X0 | Actin 1. A highly conserved protein that polymerizes to produce filaments. | O96019 | Actin-like protein 6A. Involved in transcriptional activation and repression of select genes. |
| Q8I4X0 | Actin 1. A highly conserved protein that polymerizes to produce filaments. | P61160 | Actin-related protein 2. ATP-binding component of the Arp2/3 complex |
| C0H4W3 | Probable ATP-dependent helicase PF08_0048. | Q9NS87 | Kinesin-like protein KIF15. Plus-end directed kinesin-like motor enzyme involved in mitotic spindle assembly. |
| Q8I1T8 | ATPase ASNA1 homolog. ATPase required for the post-translational delivery of tail-anchored (TA) proteins. | Q9UKX3 | Myosin-13. Fast-twitching myosin mediates the high-velocity and low-tension contractions of specific striated muscles. |
| P46468 | Putative cell division cycle ATPase. | Q9Y4C4 | Malignant fibrous histiocytoma-amplified sequence 1. Probable GTP-binding protein. |
| Q8IDR3 | Myosin-A. Myosins are actin-based motor molecules with ATPase activity. | P55039 | Developmentally-regulated GTP-binding protein 2. |
| Q8ILT5 | Protein SEY1 homolog. Probable GTP-binding protein | Q8TAI7 | GTPase RhebL1. Binds GTP and exhibits intrinsic GTPase activity. |

Another prediction is between the human protein Actin-like protein 6A (O96019) and the *Plasmodium falciparum* protein Actin (Q8I4X0). Actin is a highly conserved protein and participates in more protein-protein interactions than any known protein. It known for its ability to polymerize, forming filaments that create cross-linked networks within the cytoplasm. These networks are crucial for various cellular processes, including maintaining cell shape, enabling cell movement, and facilitating intracellular transport [1], [2]. On the other hand, Actin-like protein 6A plays a distinct role in the nucleus, where it is involved in the transcriptional activation and repression of select genes by participating in chromatin remodeling. This process is essential for regulating gene expression and ensuring that the correct genes are expressed at the right times. The predicted interaction between these two proteins suggests a potential link between cytoskeletal dynamics and gene regulation, which could provide new insights into how *Plasmodium falciparum* manipulates host cellular machinery to its advantage. This interaction was also predicted by Wutchy *et al.* in 2011, further supporting the potential significance of this connection.

The next predicted interaction involves the human protein Actin-related protein (Arp) 2 (P61160) and the *Plasmodium falciparum* protein Actin (Q8I4X0). This potential interaction between Actin and Actin-related protein 2 underscores the possibility of a functional interplay between the actin polymerization machinery of the host and the parasite which were also was predicted by Wutchy *et al.* in 2011. Actin is a highly conserved protein that polymerizes to produce filaments, forming cross-linked networks in the cytoplasm. These filaments are crucial for maintaining cell shape, enabling cell movement, and facilitating intracellular transport. Actin's role is fundamental in numerous cellular processes, including muscle contraction, cell division, and vesicle trafficking [1]. Actin-related protein 2 (P61160) is an ATP-binding component of the Arp2/3 complex. The Arp2/3 complex is essential for actin filament nucleation, which is a critical step in the formation of branched actin networks. Upon stimulation by nucleation-promoting factors, the Arp2/3 complex initiates the growth of new actin filaments, which are crucial for various cellular functions, including endocytosis, cell motility, and the establishment of cell shape. By mediating actin polymerization, Actin-related protein 2 plays a vital role in dynamically remodeling the actin cytoskeleton [2], [3].

Other predicted interaction involves the human protein Kinesin-like protein (KIF)15 (Q9NS87) and the *Plasmodium falciparum* protein Probable ATP-dependent helicase PF08_0048 (C0H4W3). PF08_0048 is the catalytic component of a chromatin remodeling complex and is considered a candidate for an effective malaria vaccine due to its epitope response in sera [4]. KIF15 is a plus-end directed kinesin-like motor enzyme involved in mitotic spindle assembly, highlighting the potential for a critical functional interaction during cell division. Similarly, the human protein Myosin-13 (Q9UKX3) and the *Plasmodium falciparum* protein ArsA-like ATPase (ASNA1) homolog (Q8I1T8) were predicted as interacting partners. Myosin-13 is a fast-twitching myosin mediating high-velocity, low-tension contractions of specific striated muscles. ATPase ASNA1 homolog is required for the post-translational delivery of tail-anchored proteins to the endoplasmic reticulum, recognizing and selectively binding the transmembrane domain of proteins in the cytosol [5].

Additional predicted interaction involves the human protein Malignant fibrous histiocytoma-amplified sequence 1 (Q9Y4C4) and the *Plasmodium falciparum* protein Putative cell division cycle ATPase (P46468). Q9Y4C4 is a probable GTP-binding protein involved in innate immunity and the inflammatory response, regulating Toll-like receptor (TLR) TLR2 and TLR4 signaling pathways (Zhong *et al.*, 2015). P46468 is part of the *Plasmodium falciparum*'s cell division machinery, underscoring the potential significance of this interaction in immune evasion and pathogen replication. The ninth predicted interaction involves the human protein Developmentally-regulated GTP-binding protein 2 (P55039) and the *Plasmodium falciparum* protein Myosin-A (Q8IDR3). Myosin-A is an actin-based motor molecule with ATPase activity, involved in intracellular movements and expressed during the asexual blood stage [7]. P55039 catalyzes the conversion of GTP to GDP through hydrolysis and may bind to RNA, playing a role in translation when hydroxylated [8]. The tenth predicted interaction involves the human protein called GTPase Ras homolog enriched in brain-like (RhebL)1 (Q8TAI7) and the *Plasmodium falciparum* protein called Suppressor of the Essential Yeast (SEY)1 homolog (Q8ILT5). RhebL1 binds GTP and exhibits intrinsic GTPase activity, activating NF-kappa-B-mediated gene transcription and promoting signal transduction [9]. Protein SEY1 homolog is involved in generating and maintaining the structure of the tubular endoplasmic reticulum network, indicating the potential for interaction in cellular structural dynamics and signaling pathways.

D. **Evaluation of optimized DF on bank customer churn dataset and MNIST image dataset**

The optimized model was trained using a bank customer churn label dataset. This dataset includes information about a bank's customers, with the target variable being a binary indicator that shows whether a customer has left the bank (closed their account) or remains a customer. The extracted dataset used in this evaluation consists of 4074 records with 14 features, namely, row number, surname, customer id, geography, credit score, age, gender, tenure, balance, number of bank products the customer is using, estimated salary, is-active-member, has-credit-card and the target variable (Exited). For this analysis, row number and customer ID were removed as they are unimportant to this evaluation. Hence only 12 features were eventually used. The result obtained is shown in Figure 3.


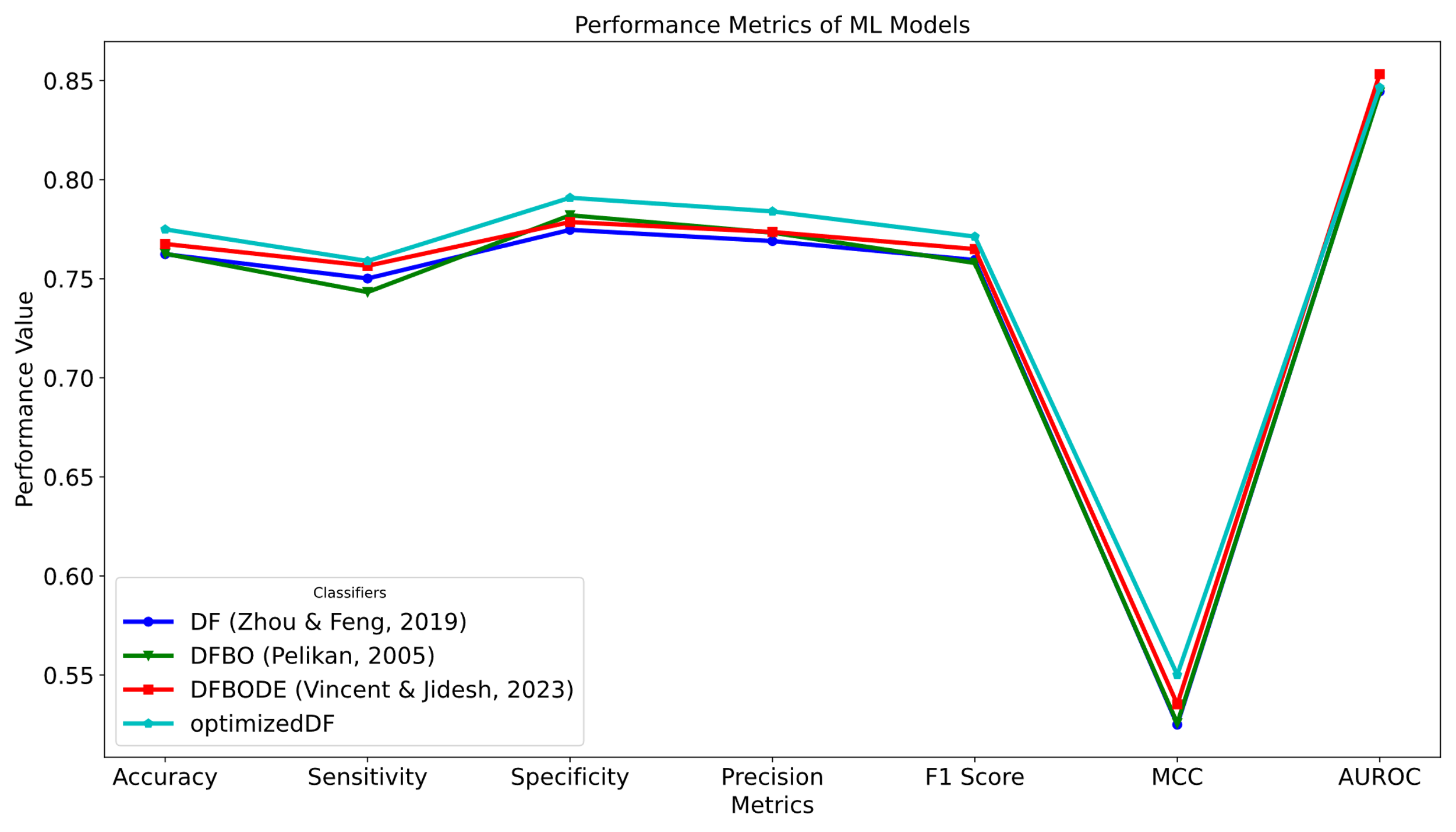


**Figure 3:** Performance comparison of optimized DF using bank customer churn dataset.

To further evaluate the performance of the optimized model, the model, alongside other optimization methods, was evaluated on image datasets. In this case, the Modified National Institute of Standards and Technology (MNIST) dataset was employed. The MNIST dataset is a subset derived from a larger dataset provided by NIST. The MNIST database of handwritten digits contains a training set of 60,000 images, consisting of 10 categories. For the purpose of this evaluation, 2 categories of images were employed. The total number of images from these 2 categories produced 11,000 samples. The results of this evaluation are in Figure 4.


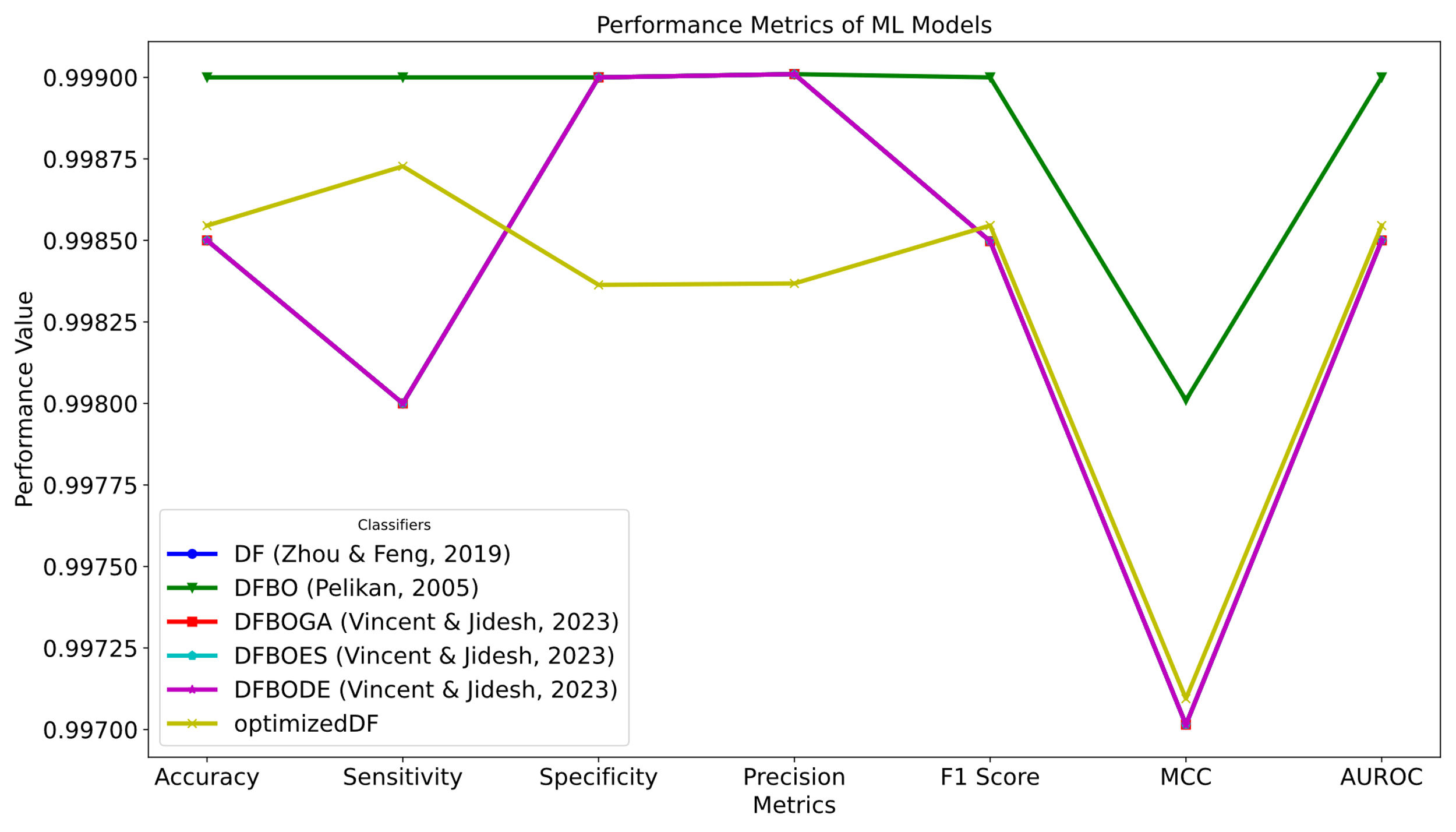


**Figure 4:** Performance comparison of optimized DF using MNIST image dataset.

E. **Evaluation of optimized DF on human-SARS-CoV-2 protein-protein interaction dataset**

The human-SARS-CoV-2 protein-protein interaction dataset used for further evaluation in this study was experimentally generated by Gordon et al. (2020) [10]. The interactions are between human proteins and 24 SARS-CoV-2 virus proteins, resulting in a total of 332 interactions. The negative interaction datasets consist of 617 non-interacting proteins, as used by Dey et al. (2020) [11]. While grouping the interactions based on host sequence similarity, all human host sequences were unique, resulting in a total of 949 group IDs. After preprocessing, a final set of 664 datasets was extracted, consisting of 332 positive (interacting) and 332 negative (non-interacting) datasets, which were subsequently used for further analysis.

Figure 5 presents the performance of the optimized DF in comparison with other optimization methods using this dataset. The results clearly demonstrate that optimizedDF outperforms DFBODE and other optimization methods across all key performance metrics. While DFBODE achieved an accuracy of 85.99%, optimizedDF surpassed this with an accuracy of 86.90%, indicating a more accurate model overall. OptimizedDF also outperformed DFBODE in terms of sensitivity, F1-score, and MCC with values of 74.40%, 85.03%, and 76.22%, respectively, compared to DFBODE’s 72.29%, 83.77%, and 74.86%. Notably, optimizedDF achieved the highest Matthews correlation coefficient (MCC) of 76.2%, surpassing DFBODE's 74.9 and demonstrating its superior ability to handle both positive and negative classes.


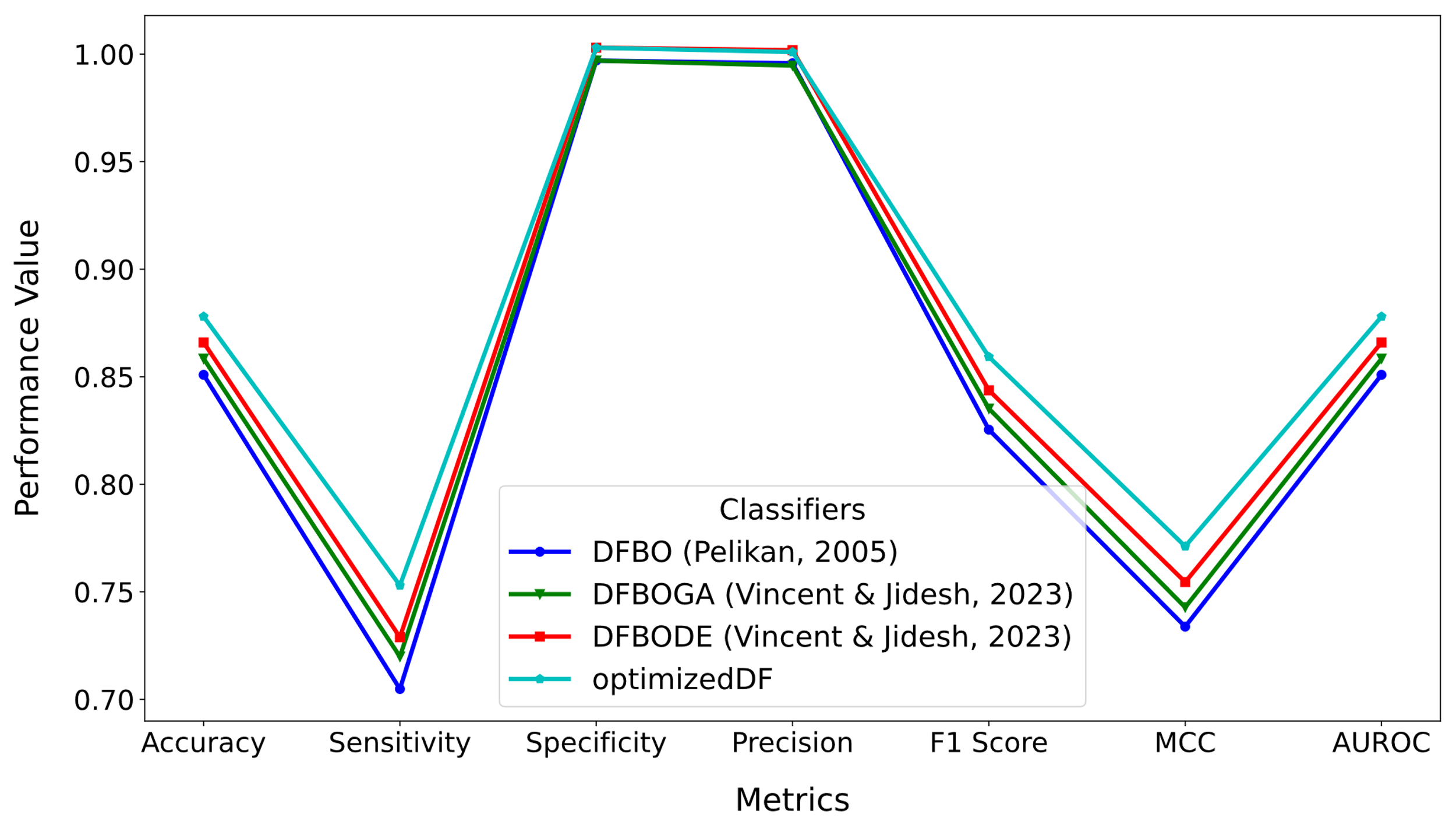


**Figure 5:** Performance comparison of optimized DF using human-SARS-CoV-2 PPI dataset.

**References**

[1] J. R. Ivanova *et al.*, “Designed Ankyrin Repeat Proteins as Actin Labels of Distinct Cytoskeletal Structures in Living Cells,” *ACS Nano*, vol. 18, no. 12, pp. 8919–8933, Mar. 2024, doi: 10.1021/ACSNANO.3C12265/SUPPL_FILE/NN3C12265_SI_004.AVI.

[2] S. M. Heissler and K. Chinthalapudi, “Structural and functional mechanisms of actin isoforms,” *FEBS J.*, 2024, doi: 10.1111/FEBS.17153.

[3] B. Lavanderos, I. Silva, P. Cruz, O. Orellana-Serradell, M. P. Saldías, and O. Cerda, “TRP Channels Regulation of Rho GTPases in Brain Context and Diseases,” *Front. Cell Dev. Biol.*, vol. 8, no. November, 2020, doi: 10.3389/fcell.2020.582975.

[4] V. Villard *et al.*, “Rapid identification of malaria vaccine candidates based on α-helical coiled coil protein motif,” *PLoS One*, vol. 2, no. 7, 2007, doi: 10.1371/journal.pone.0000645.

[5] M. J. Bloemink, J. C. Deacon, D. I. Resnicow, L. A. Leinwand, and M. A. Geeves, “The superfast Human extraocular myosin is kinetically distinct from the fast skeletal IIa, IIb, and IId isoforms,” *J. Biol. Chem.*, vol. 288, no. 38, pp. 27469–27479, 2013, doi: 10.1074/jbc.M113.488130.

[6] J. Zhong *et al.*, “MFHAS1 Is Associated with Sepsis and Stimulates TLR2/NF-κB Signaling Pathway Following Negative Regulation,” *PLoS One*, vol. 10, no. 11, pp. 1–17, 2015, doi: 10.1371/journal.pone.0143662.

[7] M. M. Alam *et al.*, “Phosphoproteomics reveals malaria parasite Protein Kinase G as a signalling hub regulating egress and invasion,” *Nat. Commun.*, vol. 6, 2015, doi: 10.1038/ncomms8285.

[8] S. Markolovic *et al.*, “The Jumonji-C oxygenase JMJD7 catalyzes (3S)-lysyl hydroxylation of TRAFAC GTPases,” *Nat. Chem. Biol.*, vol. 14, no. 7, pp. 688–695, Jul. 2018, doi: 10.1038/S41589-018-0071-Y.

[9] G. Pezzicoli, E. Filoni, A. Gernone, L. Cosmai, M. Rizzo, and C. Porta, “Playing the devil’s advocate: Should we give a second chance to mTOR inhibition in renal clear cell carcinoma? – ie strategies to revert resistance to mtor inhibitors,” *Cancer Manag. Res.*, vol. 13, no. October, pp. 7623–7636, 2021, doi: 10.2147/CMAR.S267220.

[10] D. E. Gordon *et al.*, “A SARS-CoV-2 protein interaction map reveals targets for drug repurposing,” *Nature*, vol. 583, no. 7816, pp. 459–468, 2020, doi: 10.1038/s41586-020-2286-9.

[11] L. Dey, S. Chakraborty, and A. Mukhopadhyay, “Machine learning techniques for sequence-based prediction of viral–host interactions between SARS-CoV-2 and human proteins,” *Biomed. J.*, vol. 43, no. 5, pp. 438–450, 2020, doi: 10.1016/j.bj.2020.08.003.
